# Tailored Lewis Acid Sites for High-Temperature Supported Single-Molecule Magnetism

**DOI:** 10.1021/jacs.3c02730

**Published:** 2023-06-01

**Authors:** Moritz Bernhardt, Maciej D. Korzyński, Zachariah J. Berkson, Fabrice Pointillart, Boris Le Guennic, Olivier Cador, Christophe Copéret

**Affiliations:** † Department of Chemistry and Applied Biosciences, ETH Zürich, Vladimir-Prelog Weg 1-5/10, 8093 Zürich, Switzerland; ‡ Univ Rennes CNRS, ISCR (Institut des Sciences Chimiques de Rennes), UMR 6226, 35000 Rennes, France

## Abstract

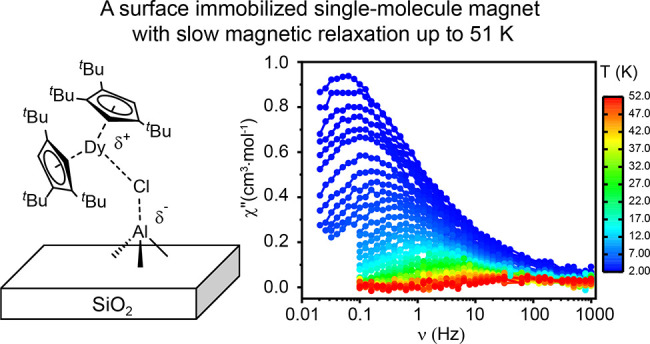

Generating or even retaining slow magnetic relaxation in surface immobilized single-molecule magnets (SMMs) from promising molecular precursors remains a great challenge. Illustrative examples are organolanthanide compounds that show promising SMM properties in molecular systems, though surface immobilization generally diminishes their magnetic performance. Here, we show how tailored Lewis acidic Al(III) sites on a silica surface enable generation of a material with SMM characteristics via chemisorption of (Cp^ttt^)_2_DyCl ((Cp^ttt^)^−^ = 1,2,4-tri(*tert*-butyl)-cyclopentadienide). Detailed studies of this system and its diamagnetic Y analogue indicate that the interaction of the metal chloride with surface Al sites results in a change of the coordination sphere around the metal center inducing for the dysprosium-containing material slow magnetic relaxation up to 51 K with hysteresis up to 8 K and an effective energy barrier (*U*_eff_) of 449 cm^–1^, the highest reported thus far for a supported SMM.

Single-molecule magnets (SMMs), compounds exhibiting slow magnetic relaxation in the absence of an external magnetic field, are anticipated to be used for data storage, quantum computing, and/or spintronics.^1−4^ Such applications require magnetic centers to be isolated on solid supports, where every site can be individually addressed.^5,6^ To date, several methodologies of SMM surface immobilization have been explored,^7−13^ focusing mostly on minimizing changes in coordination environment to avoid a decrease or loss of SMM properties.^14^ Other approaches take advantage of the direct interaction between the metal center and the surface to induce or improve SMM properties.^15−19^ A key challenge in generating magnetic remanence in supported SMMs is the multitude of possible interactions between the magnetic center and the surface, which can induce fast relaxation of the magnetic moment,^6,14,20^ and thus so far, the performances of surface deposited SMMs lag far behind molecular systems. Among such molecular systems, dysprosocenium cations—Dy(III) ions sandwiched between two substituted cyclopentadienyl moieties (Cp^R^)^−^—combine the high intrinsic magnetic moment of the lanthanide ion with a strong axial crystal field,^21−23^ resulting in high magnetic anisotropy and positioning these molecular compounds among the best SMMs.^5,24−28^ These cationic species are synthesized by the abstraction of an anionic X^–^ ligand from a neutral precursor, i.e. [(Cp^R^)_2_DyX] (Figure 1a, with X^–^ = Cl^–^, I^–^, BH_4_^–^).^24−28^ This abstraction induces a shorter distance between Dy and (Cp^R^)^−^, a wider Cp_centroid_–Dy–Cp_centroid_ angle (ω), and consequently a stronger axial crystal field leading to better SMM properties compared to its neutral precursor. Alternatively, SMM properties can also be improved by weakening the equatorial crystal fields.^29−33^

**Figure 1:**
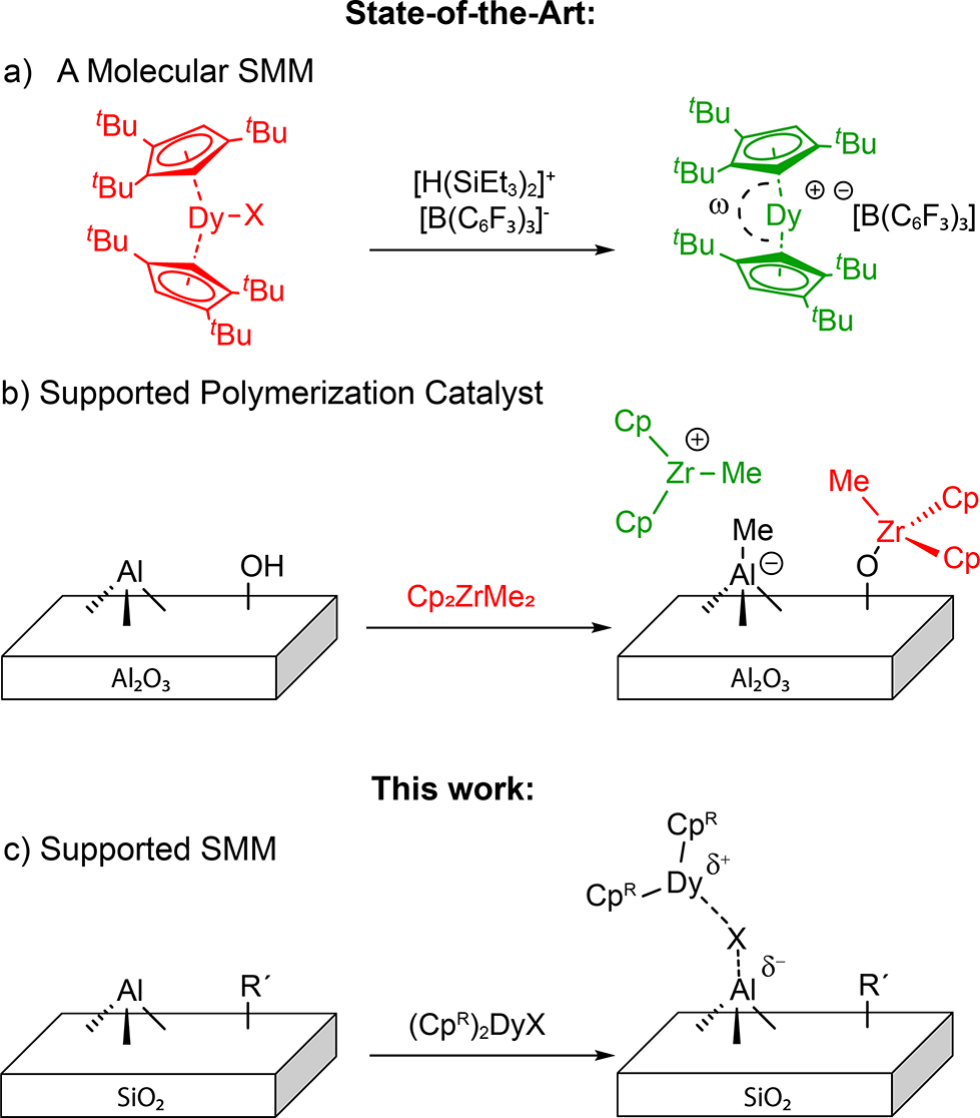
Green and red visualize cationic and neutral species, respectively. Schematic representation of ligand abstraction to form a (a) state-of-the-art dysprosocenium based SMM and (b) a cationic supported polymerization catalyst. (c) Surface immobilization toward supported SMMs.

In seeking a surface-immobilized analogue to high-performing dysprosocenium complexes, one can draw a parallel with supported polymerization catalysts based on metallocenes.^34^ A longstanding objective in the latter field has been the generation of highly active cationic metal surface sites by abstraction of one anionic ligand of the precursor by surface Lewis acid sites in the support,^35−37^ e.g. low (tri-) coordinated Al(III) centers (Figure 1b). These low-coordinate Al have been proposed to be accessible via several methods: dehydroxylation of γ-alumina^35,36^ or incorporation of aluminum sites in a rigid framework (zeolites).^38,39^ However, in many instance, neutral species are also formed due to the competitive reactions of the molecular precursor with surface OH groups.^34,40^ Supports including alkylaluminum species mitigate these problems,^41,42^ but lead to rather complex surface chemistry and the formation of multiple sites.

Herein, we report an alternative strategy by selectively generating strong Lewis acidic aluminum sites on silica while maintaining a surface largely free of OH groups. Chemisorption of (Cp^ttt^)_2_DyCl ((Cp^ttt^)^−^ = 1,2,4-tri(*tert*-butyl)cyclopentadienide)—a molecular precursor having poor SMM properties (with no magnetic hysteresis observed at 2 K),^24,43^—at these low coordinate Al sites generates a material that exhibits slow magnetic relaxation up to 51 K with an effective energy barrier of *U*_eff_ = 449 cm^–1^. Detailed chemical and computational analyses of the material and its diamagnetic Y analogue^44^ indicate a change in the coordination environment around the metal center resulting from a Lewis acid–base interaction between the surface Al sites and the chloride ligand. This interaction leads to a stronger axial crystal field, and consequently slower magnetic relaxation behavior for the Dy containing material.

We first synthesized the silica-based materials containing surface Al(III) sites via a two-step process. The first step involves grafting of a bulky trismesitylaluminum (Al(Mes)_3_) on the isolated OH groups of partially dehydroxylated amorphous silica, yielding well-defined surface (Mes)_2_Al(OSi≡) sites. In the second step, the resulting material is heated at 450 °C under high vacuum (10^–5^ mbar) to provide **Al@SiO**_**2**_ (Figures 2a and S1). This thermal treatment leads to a transfer of remaining mesityl groups from Al(III) to Si(IV) with simultaneous opening of adjacent siloxane bridges, which results in a tailored support containing isolated low-coordinate Al(III) sites in all-oxygen environments with only a minor amount of residual OH groups. This makes **Al@SiO**_**2**_ a well-suited platform for the selective abstraction of anionic ligands. Transmission Fourier-transform infrared spectroscopy (FT-IR) measurements indicate the consumption of isolated OH groups during the grafting process, which are not restored upon thermal treatment (Figure 2c). Mass balance analysis as well as ^1^H, ^13^C, and ^29^Si solid-state nuclear magnetic resonance (NMR) spectroscopy (see SI for more details, Figures S4, S5, and S10) data confirm the formation of a monografted surface species, (Mes)_2_Al(OSi≡), before heat treatment. The heat-treated material contains primarily Al(OSi≡)_3_ sites along with a small amount (around 10%) of remaining mesityl-aluminum moieties. Wideline solid-state ^27^Al NMR spectra (Figure 3a) of **Al@SiO**_**2**_ indicate the presence of two main Al(III) sites, each exhibiting different quadrupolar coupling constants (*C*_Q_) of ca. 14 (site I, violet) and 22 MHz (site II, green), which are assigned to two types of surface Al sites in distorted, tetrahedral oxidic environments. The presence of strong Lewis acid sites on the support is established by exposing the material to ^15^N pyridine and recording its solid-state ^15^N{^1^H} CP-MAS spectrum (Figure S6) which displays an intense signal at 252 ppm along with a weaker signal at 216 ppm, consistent with the presence of strong Lewis acid sites along with a small amount of residual acidic silanols (not observed by IR). Notably, the ^27^Al NMR spectrum of this material (Figure 3b) indicates that site I is not affected by adsorption of pyridine, while site II shows a strong decrease of *C*_Q_ from 22 to 15 MHz, indicating that only site II is prone to coordinate Lewis bases, suggesting that it is a highly distorted tetrahedral site consistent with its large *C*_Q_ value.

**Figure 2:**
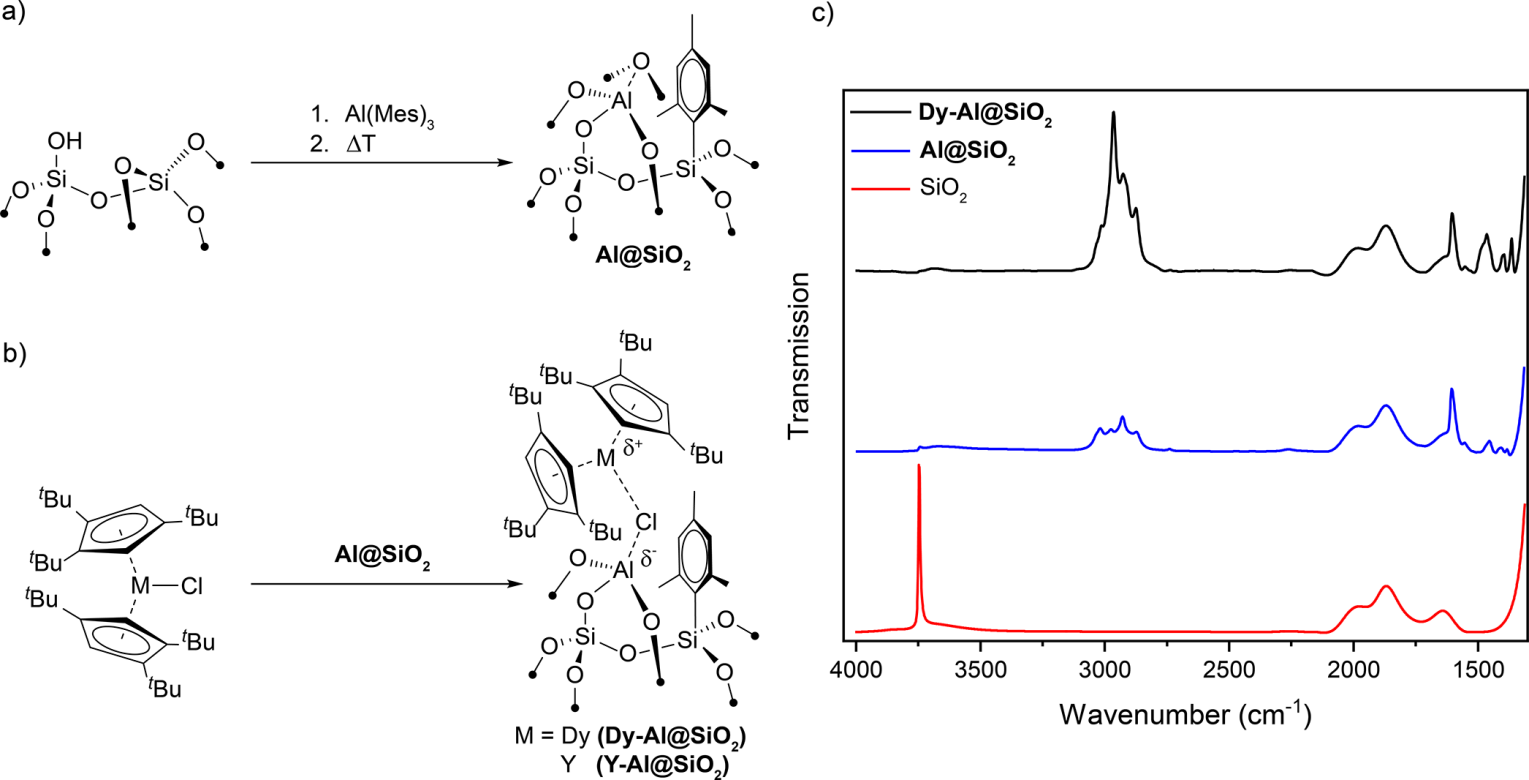
(a) Synthetic route to **Al@SiO**_**2**_ and (b) surface deposition of [(Cp^ttt^)_2_MCl] yielding **M-Al@SiO**_**2**_ (a possible surface species is displayed). (c) Comparison of FT-IR spectra of SiO_2_ (red), **Al@SiO**_**2**_ (blue), and **Dy-Al@SiO**_**2**_ (black).

**Figure 3:**
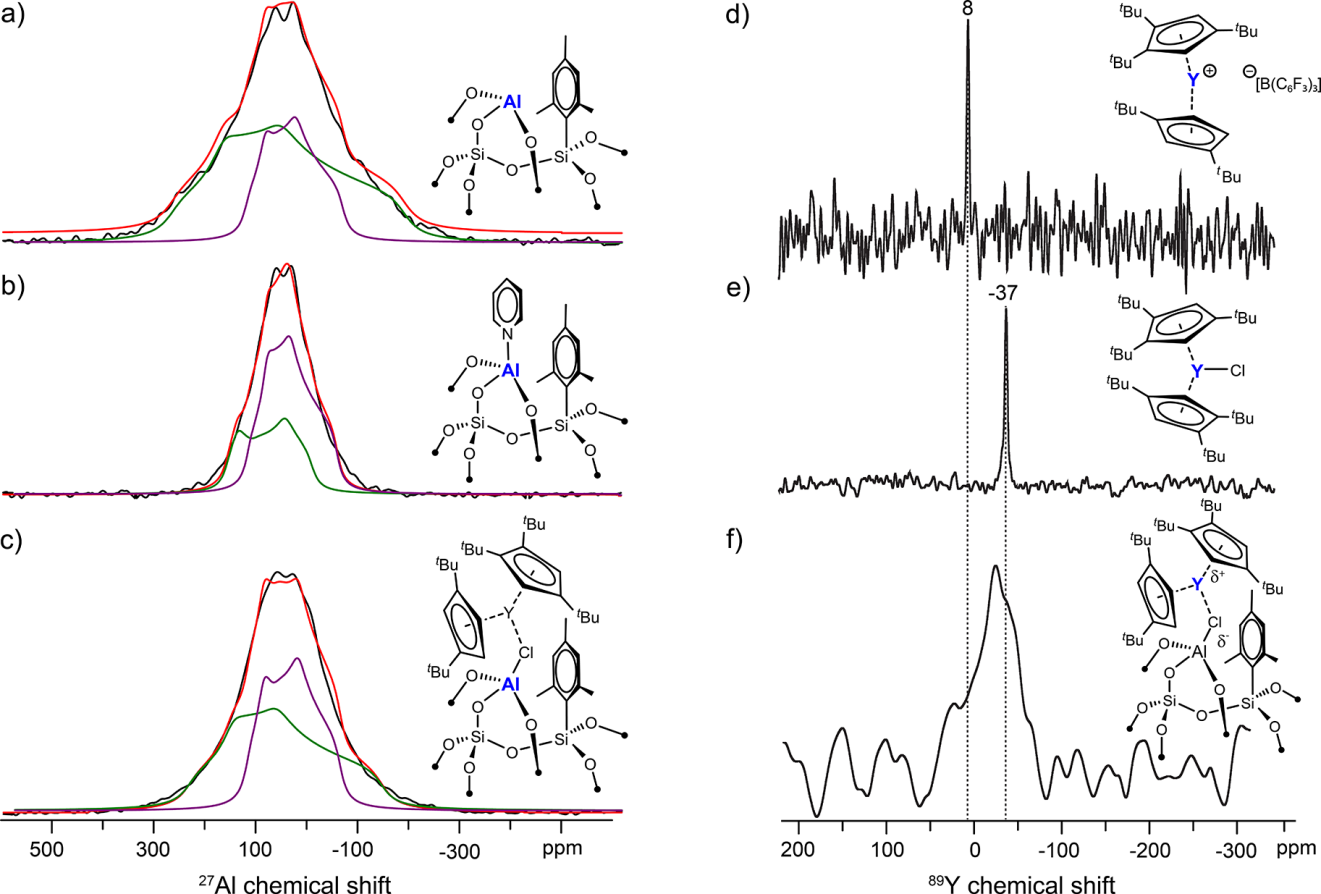
Solid-state static ^27^Al WURST-QCPMG spectra^45^ (black) of **Al@SiO**_**2**_ (a) before and (b) after exposure to ^15^N-labeled pyridine and of (c) **Y-Al@SiO**_**2**_, along with line shape simulations (red) and spectral deconvolutions (site I: violet, site II: green). Spectra were acquired at 20.0 T and 265 K. Solid-state ^89^Y{^1^H} CP-MAS NMR spectra of (d) [(Cp^ttt^)_2_Y]^+^[B(C_6_F_5_)_4_]^−^, (e) (Cp^ttt^)_2_YCl, and (f) **Y-Al@SiO**_**2**_, acquired at 100 K, 8 kHz MAS, 9.4 T, and using DNP-enhanced measurement conditions^46^ for (f) (see Supporting Information for experimental details).

Next, **Al@SiO**_**2**_ was combined with (Cp^ttt^)_2_DyCl or its diamagnetic analogue (Cp^ttt^)_2_YCl to yield the corresponding materials **Dy-Al@SiO**_**2**_ and **Y-Al@SiO**_**2**_ (Figure 2b). FT-IR measurements of these materials (Figures 2c and S7) show an increase of the relative intensity and a changed intensity distribution of C–H stretching modes (3050–2850 cm^–1^) compared to the rare-earth free material with features that are in line with those of the molecular precursors.^24,25^ The IR data and the low M/Al molar ratios (ca. 0.25) for **Dy-Al@SiO**_**2**_ and **Y-Al@SiO**_**2**_ suggest a successful surface deposition of the precursor most likely via interaction of the chloride ligand with the most reactive Lewis acidic sites. In order to understand the nature of the precursor–support interaction, we investigated **Y-Al@SiO**_**2**_ via solid-state ^1^H and ^13^C magic-angle-spinning (MAS) NMR. The data confirm the presence of (Cp^ttt^)^−^ and mesityl moieties supporting a successful surface deposition of the precursor (Figures S8 and S9). Furthermore, ^27^Al NMR shows that the *C*_Q_ value of Al site II decreases from 22 to 19 MHz upon reaction with (Cp^ttt^)_2_YCl (Figure 3c), pointing to an interaction of Al site II with the Y precursor while site I remains unperturbed, paralleling the observations upon pyridine adsorption. This interaction is corroborated by solid-state ^89^Y{^1^H} CP NMR spectra acquired with sensitivity enhanced by dynamic nuclear polarization (DNP),^46,47^ which show a broad ^89^Y signal spanning ca. −20 to −50 ppm (Figure 3f). The broad linewidth indicates a distribution of surface species, likely arising from the heterogeneity of the amorphous support and a distribution of Y–Cl bond lengths and local environments. Compared to the ^89^Y signal positions of the cationic [(Cp^ttt^)_2_Y]^+^[B(C_6_F_5_)_4_]^−^ (8 ppm) and the neutral (Cp^ttt^)_2_YCl (−37 ppm) (Figure 3d and e), **Y-Al@SiO**_**2**_ likely retains a (partial) Y–Cl bond. Together, the solid-state ^89^Y and ^27^Al NMR analyses confirm an interaction of the Y–Cl moieties with Al surface sites, overall leading to a distribution of elongated Y–Cl distances.

The magnetic properties of **Dy-Al@SiO**_**2**_ were evaluated using alternating current (AC) and direct current (DC) experiments. The out-of-phase (χ′′(ν), with ν denoting the oscillating field frequency) component of the AC magnetic susceptibility exhibits maxima between 2 and 51 K (Figure 4a) under an oscillating field in the absence of an applied static magnetic field. Over the whole measured range, χ′′ shows a temperature dependence visualized by the shift of the maxima upon increasing temperatures. In contrast, (Cp^ttt^)_2_DyCl does not exhibit slow relaxation characteristics under these conditions.^24^ To gain more insights into the specific relaxation times and mechanisms, the AC susceptibility data in the 2 to 49 K range were fitted using the extended Debye model showing a wide distribution of relaxation times with an α_max_ of 0.56 (Table S1) resulting from a distribution of magnetic sites on the surface, consistent with the ^89^Y NMR data and other reports on surface deposited SMMs.^17,19^ The extracted specific relaxation times τ for each temperature can be expressed with the two component fit of τ^–1^ = τ_0_^–1^*e*^–(*U*_eff_)/(*k*_B_*T*)^ + *CT*^*n*^ (Figure 4b), in which the first term describes the Orbach mechanism (over an effective energy barrier *U*_eff_, *k*_B_ representing the Boltzmann constant) and the second term describes the Raman process (*C* is the Raman coefficient and n the Raman exponent). To the best of our knowledge, **Dy-Al@SiO**_**2**_ not only shows slow relaxation at the highest reported temperature but also has the highest reported *U*_eff_ = 449 cm^–1^ (τ_0_ = 5.24 × 10^–9^ s, *C* = 9.3 × 10^–2^ s^–1^ K^–*n*^ and *n* = 1.75) for an immobilized SMM (see Supporting Information (SI) for more details, Figure S13 and Table S2). The field-dependency of the magnetization was investigated in the range of an applied field between −20 to +20 kOe using a field sweep rate of 16 Oe s^–1^ between 2 and 8 K (Figure 4c). Under these conditions, the hysteresis loops remain open up to 8 K. At 2 K a remnant magnetization of 0.9 Nβ and a coercive field of 354 Oe was found, further demonstrating the SMM character of **Dy-Al@SiO**_**2**_. The opening of the hysteresis loop supports the hypothesis that the interaction of (Cp^ttt^)_2_DyCl with **Al@SiO**_**2**_ induces SMM behavior.

To further corroborate these results, the dependence of magnetic properties of model structures as a function of the Dy–Cl distance were investigated computationally using an *ab initio* CASSCF/RASSI-SO/SINGLE_ANISO approach (see SI for more details). As expected, the energy splitting between the two lowest Kramers doublets is driven by the Dy–Cl bond length, with longer bonds yielding higher energy spacings. Note that elongation of the Dy–Cl bond leads to wider ω angle and shorter Dy–Cp_centroid_ distance consistent with the improved SMM properties (see SI, Figures S16–S22 and Tables S3–S6). Comparison of the calculated *χT* and magnetization curves at 2 K (Figures S14 and S15) of all the model structures with the measured data suggest an increase in Dy–Cl bond length from 2.54 Å in (Cp^ttt^)_2_DyCl to around 2.6–3.1 Å in **Dy-Al@SiO**_**2**_.

**Figure 4:**
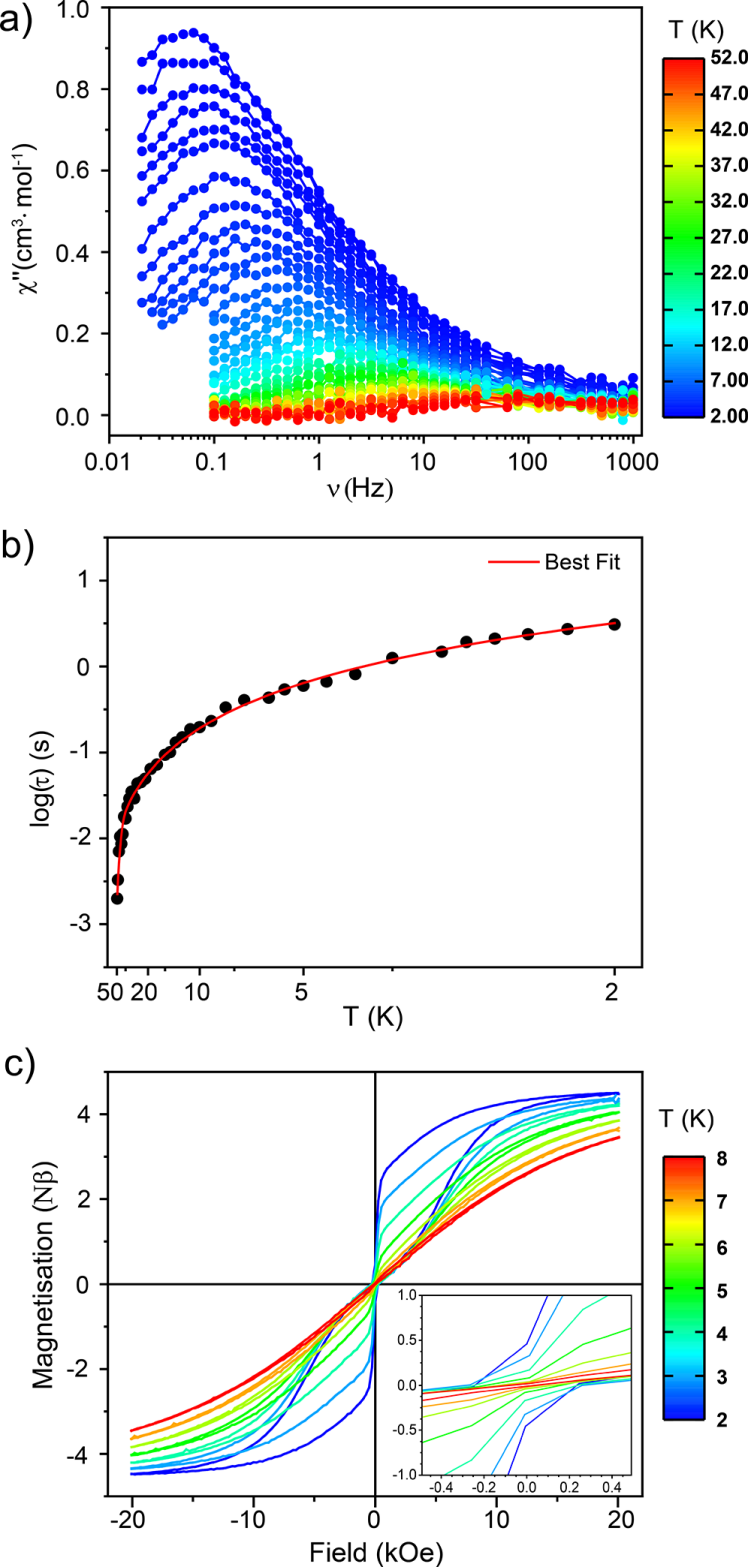
Magnetic characterization of **Dy-Al@SiO**_**2**_: (a) frequency dependence of the out-of-phase component of the AC susceptibility measured in zero external DC field between 2 and 51 K using a 3 Oe amplitude, (b) temperature dependence of the relaxation time between 2 and 49 K where the red line is the best fit using the parameters in the text, and (c) hysteresis curves recorded between 2 and 8 K with 16 Oe s^–1^ field sweep rate. Inset shows a zoom of the zero-field region.

In conclusion, this work shows that selectively formed Lewis acidic Al(III) surface sites can be used to immobilize (Cp^ttt^)_2_MCl via Lewis acid–base interaction. The resulting change in coordination environment engenders SMM properties in the case of **Dy-Al@SiO**_**2**_. These magnetic properties are unprecedented for a surface immobilized SMM with slow magnetic relaxation up to 51 K and hysteresis observed up to 8 K. These results open up a novel path to surface immobilization of SMMs and further motivate approaches to generate fully charge separated species on surfaces.

## Supporting Information Available

Experimental details, Solid-state NMR, FT-IR and elemental analyses, magnetic characterization, and computational data. (PDF)
