# Supplementary material for: Tailored Lewis Acid Sites for High-Temperature Supported Single-Molecule Magnetism
Source: J Am Chem Soc. 2023 Jun 1;145(23):12446–51. doi: 10.1021/jacs.3c02730 (PMC10273314; doi:10.1021/jacs.3c02730)
Supplement: Supplementary file 1 — ja3c02730_si_001.pdf [file ja3c02730_si_001.pdf]

Supporting Information: Tailored Lewis Acid Sites for High-Temperature Supported Single-Molecule Magnetism

Moritz Bernhardt[a],† Maciej D. Korzyński[a],† Zachariah J. Berkson,† Fabrice Pointillart,‡ Boris Le Guennic,*,‡ Olivier Cador,*,‡ and Christophe Copéret*,†

†*Department of Chemistry and Applied Biosciences, ETH Zürich, Vladimir-Prelog Weg 1-5/10, 8093 Zürich, Switzerland*

‡*Univ Rennes, CNRS, ISCR (Institut des Sciences Chimiques de Rennes), UMR 6226, 35000 Rennes, France*

E-mail: boris.leguennic@univ-rennes.fr; olivier.cador@univ-rennes.fr; ccoperet@ethz.ch

[a] M.B. and M.D.K. contributed equally to this work.

General information

Dicyclopentadiene, 2-bromo-2-methylpropane (for synthesis), sodium hydride (60% dispersion in mineral oil), potassium bis(trimethylsilyl)amide (95%), diethyl ether (ACS Reag., ≥99.8%), n-butyllithium solution (1.6M in hexane) and pentane (HPLC, ≥99.0%) were obtained from Sigma Aldrich. Eicosane (99%) was purchased from Acros Organics. Tetrahydrofuran (LiChrosolv® for liquid chromatography) was obtained from Merck. Toluene (≥99.8%) was purchased from Fisher Chemical. Dibenzo-18-crown-6 (purum, ≥99.0%) was obtained from Fluka Chemie. Dysprosium(III) chloride (anhydrous, 99.9%) was purchased from Strem Chemicals, Inc. (STR). Aluminum chloride (anhydrous, 99.99%) and mesityl bromide (99%) were obtained from abcr GmbH. Benzene-*d6* (99.5% D) was purchased from Eurisotop. Ultrapure water (18.2 MΩ·cm) was obtained using Synergy® purification system. Toluene, diethyl ether, benzene, THF and pentane were dried using the commercial MBraun SPS-800 solvent purification system, degassed via three freeze-pump-thaw cycles. All solvents were stored over 4 Å molecular sieves under argon atmosphere for several days prior to use. Benzene-*d6* were distilled from sodium in presence of benzophenone and stored over 4 Å mole sieves under argon atmosphere. 1,3,5-Tri(*tert*-butyl)cyclopentadiene[S1] ($Cp^{ttt}H$), KCp^{ttt},[S2] $Cp^{ttt}{}_2MCl$ (M=Y,Dy),[S3] $[(Cp^{ttt})_2Y][B(C_6F_5)_4]$,[S3] MesLi,[S4] $AlMes_3$[S5] and SiO_2[S6] were prepared according to the literature procedures. Unless stated otherwise, all manipulations were performed using standard Schlenk techniques or in MBraun gloveboxes with argon as an inert gas (<0.1 ppm O_2 and H_2O).

Transmission Fourier-transformed infrared spectroscopy (FT-IR) was collected using a Brucker ALPHA II spectrometer used in an argon filled glovebox. The spectrometer is equipped with a KBr beamsplitter, a temperature stabilized DTGS detector and a transmission quick snap module. The data were collected in the range of 4000 to 400 cm^{-1} with a resolution of 4 cm^{-1} averaging over 2 scans.

Elemental analyses were performed by Mikroanalytisches Labor Pascher, Remagen, Germany.

Synthetic procedures

Preparation of $(Mes)_2Al(OSi\equiv)$.

In the glovebox SiO_2 (0.35 mmol OH/g, 1.9494 g, 0.68 mmol OH) was suspended in benzene (17.5 mL). To this white suspension a colorless solution of Mes_3Al (262.3 mg, 0.68 mmol) in benzene (4 mL) was added in a drop-wise fashion with gentle stirring (100 rpm). The suspension was left stirring for 3 hours at room temperature. The liquid phase was removed and the solid materials washed 5 times with 5 mL portions of fresh benzene. The obtained white solids were dried under high vacuum (10^{-5} mbar) at room temperature for 12 hours. EA data for representative sample: Al, 0.78 %; C, 6.48 %; H, 0.65 %.

Preparation of Al@SiO_2.

$(Mes)_2Al(OSi\equiv)$ (200 mg) was placed in a quartz reactor inside of a glovebox. The material was placed under high vacuum (10^{-5} mbar) and heated to 450 °C over the period of 8 hours and kept at the temperature for the subsequent 8 hours. After cooling down of the reactor, the resulting yellow material was transferred and stored in the glovebox. EA data for representative sample: Al, 0.84 %; C, 3.27 %; H, 0.29 %.

Procedure for natural abundance and ^{15}N pyridine adsorption.

In the glovebox 50 mg of the desired material was placed in a sealed reactor. The reactor was evacuated and exposed to the autogenous vapor pressure of pyridine for 10 minutes at room temperature. Following pyridine exposure, the material was dried under high vacuum (10^{-5} mbar) for 12 hours and stored in the glovebox.

Preparation of Dy-Al@SiO_2.

Al@SiO_2 (120 mg, 37 µmol Al) was placed in a Schlenk flask equipped with a magnetic stir bar and 3 ml benzene were added. A solution of $(Cp^{ttt})_2DyCl$ (24 mg, 36 µmol) in 7 ml benzene was added drop-wise with slow stirring (100 rpm). The reaction mixture was stirred

over-night, the supernatant removed and the solids washed 3 times with 5 ml fresh benzene. The product was dried under high vaccum (10^{-5} mbar) for 5 h. EA data for a representative sample: Dy, 1.14 %; Cl, 0.26 %; Al, 0.76 %; C, 6.05 %; H, 0.68 %.

Preparation of Y-Al@SiO$_2$.

Al@SiO$_2$ (170 mg, 53 µmol Al) was placed in a Schlenk flask equipped with a magnetic stir bar and 3.5 ml benzene were added. A solution of $(Cp^{ttt})_2YCl$ (29.0 mg, 53 µmol) in 7 ml benzene was added drop-wise with slow stirring (100 rpm). The reaction mixture was stirred over-night, the supernatant removed and the solids washed 3 times with 5 ml fresh benzene. The product was dried under high vacuum (10^{-5} mbar) for 5 h. EA data for a representative sample: Y, 0.65 %; Cl, 0.26 %; Al, 0.72 %; C, 6.00 %; H, 0.70 %.

Solid-state NMR analyses

All materials were prepared for solid-state NMR analyses under dry and oxygen-free argon atmosphere in an MBraun or GS glovebox equipped with a purifier unit (<0.5 ppm O_2 and H_2O). 3.2 mm zirconia or sapphire NMR rotors and 3.2 mm zirconia NMR drive caps were dried overnight at 150 °C and introduced into the glovebox while hot, and all plastic NMR rotor caps and packing tools were introduced into the glovebox overnight under vacuum to avoid contamination with air or water. After sample packing, the NMR rotors were capped and sealed under argon in vials with screw-cap tops and silicone/PTFE gaskets, which were further sealed with electrical tape. The NMR rotors were transferred under Ar to the NMR spectrometer, rapidly inserted into the MAS NMR probe head, and spun with dry N_2 gas. For the measurements at 14.1 T and 9.4. T, the MAS NMR probe head was pre-cooled to ca. 100 K (measured in the gas stream in the MAS spinning housing) prior to pneumatic insertion of the MAS NMR rotors. For room-temperature measurements at 16.4 T, the organometallic compound **Y-Al@SiO$_2$** was sealed with a silicone plug to further prevent contamination with air or water; this resulted in an additional background signal in the ^{1}H, ^{13}C, and ^{29}Si NMR spectra of this material. Chemical shifts were referenced to reported chemical shift values for ^{15}N,^{13}C-glycine for ^{1}H, ^{13}C, and ^{15}N, 1 M $Al(NO_3)_3$ solution for ^{27}Al, tris(*tert*butoxy)silanol for ^{29}Si, and YCl_3 for ^{89}Y.

Solid-state ^{15}N{^{1}H} CPMAS NMR spectra of ^{15}N pyridine-adsorbed materials were acquired on a Bruker Avance III 600 MHz (14.1 T) DNP NMR spectrometer equipped with a low-temperature 3.2 mm double-resonance MAS cryo-probe operating at Larmor frequencies of 599.900 and 60.803 MHz for ^{1}H and ^{15}N, respectively. ^{15}N{^{1}H} cross-polarization (CP) magic angle spinning (MAS) NMR spectra were acquired with ^{15}N{^{1}H} contact times of 3 ms, at 8 kHz MAS, and with 100 kHz SPINAL-64 ^{1}H heteronuclear decoupling[S7] during the acquisition periods.

Solid-state ^{1}H, ^{13}C, ^{13}C{^{1}H} and ^{29}Si{^{1}H} spectra were acquired on a 700 MHz (16.4 T) Bruker NMR spectrometer equipped with a broadband 3.2 mm HX MAS probe head and

operating at Larmor frequencies of 139.086, 176.061 and 700.135 MHz for ^{29}Si, ^{13}C, and ^{1}H, respectively. Spectra were acquired at 10 kHz MAS and 295 K. ^{29}Si{^{1}H} CP spectra were acquired with ^{29}Si-^{1}H contact times of 8 ms using Carr-Purcell-Meiboom-Gill (CPMG) detection[S8] to enhance sensitivity. 16 echoes were acquired during the detection period with rotor-synchronized echo durations of 12 rotor periods and 2 rotor period echo delay. CP-CPMG spectra were processed by magnitude correction. The ^{13}C{^{1}H} CPMAS NMR spectra were acquired with ^{13}C-^{1}H contact times of 0.2 or 2 ms.

Solid-state ^{89}Y{^{1}H} and ^{13}C{^{1}H} DNP and LTMAS CP NMR spectra were acquired on a Bruker Avance III 40 MHz (9.4 T) DNP NMR spectrometer equipped with a low-temperature 3.2 mm double-resonance MAS cryo-probe operating at Larmor frequencies of 400.610, 100.741 and 19.629 MHz for ^{1}H, ^{13}C, and ^{89}Y, respectively. ^{89}Y{^{1}H} CP-LTMAS spectra of $(Cp^{ttt})_2YCl$ and $[(Cp^{ttt})_2Y]^+[B(C_6F_5)_4]^-$ were acquired at 8 kHz MAS with ^{89}Y{^{1}H} contact times of 30 ms. For the DNP-enhanced ^{89}Y{^{1}H} and ^{13}C{^{1}H} measurements of **Y-Al@SiO$_2$**, the material was impregnated in a glovebox with a solution of 16 mM TEKPol biradical[S9] in 1,1,2,2-tetrachloroethane solvent (1.5 µL per mg of compound) and packed into a sapphire NMR rotor with a zirconia drive cap. DNP-enhanced spectra were acquired under 263 GHz continuous wave microwave irradiation at 5 W generated by a Bruker klystron unit. DNP enhancements measured by comparing the microwave on/off ^{1}H spectra were ca. x50. ^{13}C{^{1}H} DNP-CPMAS spectra were acquired at 10 kHz MAS with contact times of 0.2 or 2 ms. ^{89}Y{^{1}H} DNP-CPMAS spectra were acquired at 8 kHz MAS with ^{89}Y{^{1}H} contact times of 30 ms. All ^{89}Y{^{1}H} DNP-CPMAS and LTMAS spectra were acquired using spin-echo detection to minimize the effects of acoustic ringing, with a rotor synchronized echo delay of 2 rotor periods.

All ^{13}C{^{1}H}, ^{29}Si{^{1}H}, ^{15}N{^{1}H} and ^{89}Y{^{1}H} CPMAS spectra were acquired with 100 kHz SPINAL-64 ^{1}H heteronuclear decoupling[S7] during the acquisition periods. Generally, relaxation delays for CP spectra were set to ca. 1.4 times the longest measured ^{1}H spin-lattice T_1 (or T_{DNP}) relaxation time measured by ^{1}H saturation recovery.

The ^{27}Al NMR spectra were acquired on a 850 MHz (20.0 T) widebore Bruker NMR spectrometer equipped with a broadband 3.2 mm HXY MAS probe head operating at a ^{27}Al Larmor frequency of 221.496 MHz. The samples were flame sealed under high vacuum into quartz tubes with 4 mm outer diameter and placed into the NMR probe. Sample temperature was regulated at 270 K using a Bruker VTU unit. Spectra were acquired under static conditions using a wideline WURST-QCPMG[S10,S11] pulse sequence with a 200 kHz adiabatic frequency sweep (high-to-low and low-to-high frequency sweeps were both acquired and co-added) and a 0.05 s relaxation delay. 50 echoes were acquired in the acquisition period (0.02 s acquisition time) with echo durations of 0.3 ms and a 0.1 ms inter-echo delay. Absorptive lineshapes were reconstructed by extraction, Fourier transform, and co-addition of the first 30 echoes. WURST-QCPMG spectra were processed by magnitude correction.

Characterization of the support

Treatment of SiO_2 with 1 eq of Mes_3Al in benzene at room temperature led to no visible changes in the reaction mixture (Fig. S1). However, liquid phase ^1H NMR spectrum of this process revealed a release of mesitylene, indicating a protonation of the organometallic precursor. Investigation of transmission FT-IR spectrum of the obtained $(Mes)_2Al(OSi\equiv)$ (Fig. S2) confirms the complete consumption of the silanol moieties, evidenced by the disappearance of sharp feature at 3747 cm^{-1} present in the starting SiO_2. The spectrum also revealed the emergence of a sharp feature (1603 cm^{-1}) that can be ascribed to mesityl fragment as it is present in free mesitylene.[S12] The presence of such features points to immobilization of an organometallic fragment on the surface of silica. Elemental analysis (EA) confirms this supposition with the surface species stoichiometry corresponding to Mes:Al molar ratio of 2:1. It is well established that the initial grafting of alkylalumminum species on the surface of silica is typically immediately followed by opening of the reactive siloxane bridges to form Al–O linkages with a concomitant transfer of the alkyl moiety to neighboring Si(IV) sites.[S13] To evaluate if treatment with arylaluminum reagent, Mes_3Al, leads to similar outcome, we treated $(Mes)_2Al(OSi\equiv)$ with an excess of TBOSOH (protonating agent that is a molecular analog of surface silanol groups) in benzene-*d6*. This treatment resulted in a quantitative release of free mesitylene into the liquid phase as judged from liquid-state ^1H NMR spectroscopy. As only the Mes groups bound to Al(III) would undergo protonation (Si(IV)-bound Mes moieties would not be affected) the obtained results suggest that all Mes moieties are bound to Al(III). The absence of Si-Mes moieties in $(Mes)_2Al(OSi\equiv)$ is further corroborated by solid-state $^{29}Si\{^1H\}$ CP-CPMG NMR spectra (Fig. S10a). A single broad ^{29}Si signal is observed at -107 ppm in the ^{29}Si NMR spectrum of $(Mes)_2Al(OSi\equiv)$, which can be attributed to Si tetrahedral in the silica support,[S14] with no evidence for migration of Mes moieties to silicon sites during grafting. While in theory the presence of Lewis acidic $(Mes)_2Al(OSi\equiv)$ sites could allow for the desired chloride abstraction from $(Cp^{ttt})_2DyCl$ to occur, the combined steric bulk of Mes and $(Cp^{ttt})^-$ fragments could hinder the inner sphere

ligand exchange. Furthermore, the presence of Al(III)-bound labile Mes groups could lead to adventitious ligand metathesis with $(Cp^{ttt})^-$ bound to Dy(III) and result in compromised SMM properties. To address both of these issues we decided to thermally induce the transfer of Mes groups to Si(IV). Heating of $(Mes)_2Al(OSi\equiv)$ at 450 °C under high vacuum resulted in a change of material color from white to yellow (Fig. S1). The transmission FT-IR spectrum of the resulting **Al@SiO$_2$** does not appreciably differ from that of the starting material (Fig. S2). The feature at 1603 cm^{-1} is still present (slightly broader than in $(Mes)_2Al(OSi\equiv)$), indicating that Mes fragments persisted during the thermal treatment. Most notably, the heating does not lead to reemergence of OH groups. Thus, no deleterious activity associated with Brønsted acidic sites is to be expected when contacting the $(Mes)_2Al(OSi\equiv)$ with $(Cp^{ttt})_2DyCl$. EA analysis reveals a lower Al:Mes ration of 1:1 compared to the non-heat treated material. We hypothesize that during the Mes transfer from Al to Si part of the mesityl is removed from the surface. To confirm that the transfer of Mes groups to Si(IV) has occurred the TBOSOH protonation experiment was repeated. In this instance only 8 % of surface bound mesitylene has been released from the material showing that only a fraction of Mes moieties remain bound to Al(III). While heating of the materials to higher temperature could lead to complete removal of Mes–Al fragments, our test experiments indicated that a more complex decomposition mechanisms begin at 500 °C. Finally, inspection of ^{29}Si CP-CPMG NMR spectrum of **Al@SiO$_2$** (Fig. S10b) provides a clear evidence for the transfer of Mes groups to Si(IV) sites. In addition to the Q_4 sites seen in the starting material, an additional signal (δ_{iso} = -65 ppm) can be detected that is characteristic of Si(IV) surrounded by three oxygens and one hydrocarbyl substituent.[S14] In conclusion, **Al@SiO$_2$** contains exposed surface Al(III) sites in all oxygen environment.

To gauge the strength of Lewis acid sites present on the surface of **Al@SiO$_2$**, we employed the common probe molecule, pyridine. Spectroscopic features of this molecule are sensitive to the nature and strength of acidic sites present. First, we exposed **Al@SiO$_2$** to natural abundance pyridine vapors and evaluated the transmission FT-IR spectrum of the resulting

material (Fig. S3). The characteristic 8a mode of the neat pyridine is significantly shifted following the interaction with the surface Al(III) sites and can be observed at 1620 cm^{-1} (Fig. S3 inset). Such shift indicates strongly Lewis acidic character, almost on par with the most acidic sites in supports such as γ-alumina and silica-alumina (1622 cm^{-1}).[S15] Furthermore, no appreciable features can be observed at 1635 cm^{-1} and 1545 cm^{-1} (characteristic for pyridinium ion[S16]) confirming that **Al@SiO$_2$** has almost no Brønsted acidic character. Indeed, solid-state $^{15}N\{^{1}H\}$ CPMAS NMR spectra of $(Mes)_2Al(OSi\equiv)$ before and after thermal treatment at 450 °C (Fig. S6) confirm the presence of a single type of Lewis acid site in both materials. The ^{15}N chemical shift of ^{15}N-pyridine adsorbed on $(Mes)_2Al(OSi\equiv)$ is observed at 257 ppm, considerably shifted from the signal of free pyridine at 307 ppm,[S17] consistent with coordination to a strong Lewis acid site. After thermal treatment, the signal shifts further to 252 ppm, indicating a slight increase in the Lewis acidity of the Al centers. A weak signal at 216 ppm is also detected, which might arise from the presence of a small fraction of Brønsted acid sites generated on thermal rearrangement of the support surface.

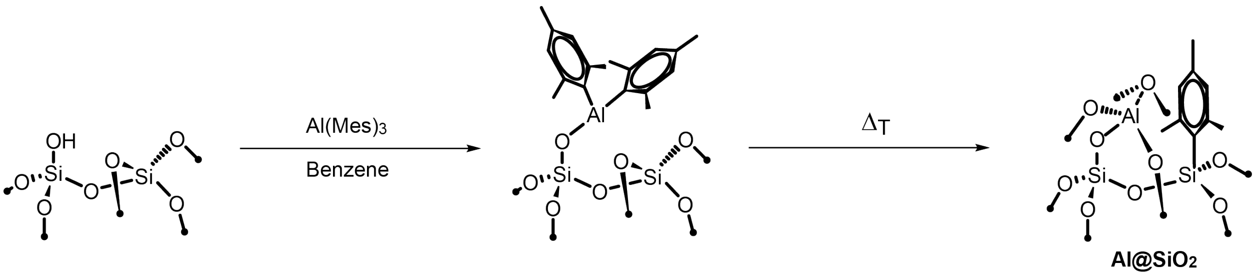

Figure S1: Synthetic steps to form $(Mes)_2Al(OSi\equiv)$ and **Al@SiO$_2$**(for both materials a possible surface species is displayed).

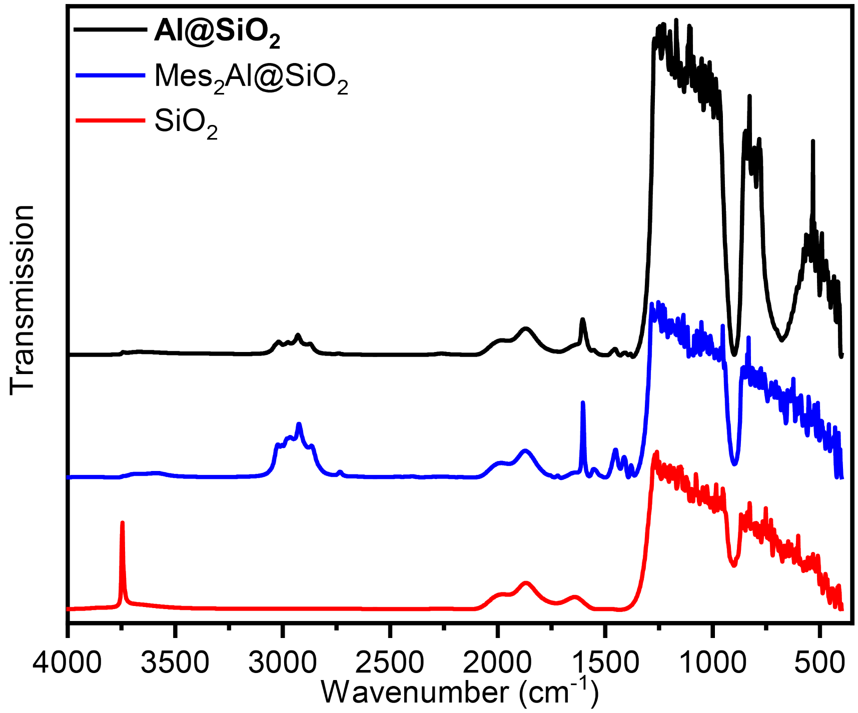

Figure S2: Comparison of transmission FT-IR spectra of starting SiO_2 (red), $(Mes)_2Al(OSi\equiv)$ (blue) and **Al@SiO$_2$** (black).

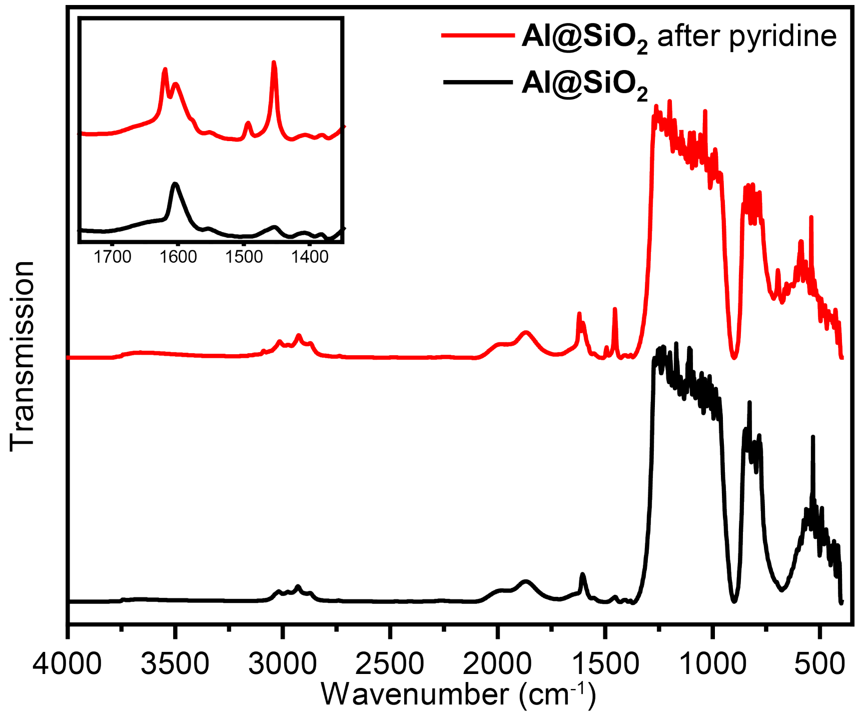

Figure S3: Comparison of transmission FT-IR spectra of **Al@SiO$_2$**(black) before and after exposure to natural abundance pyridine (red). Inset shows magnification of the relevant region of the spectrum.

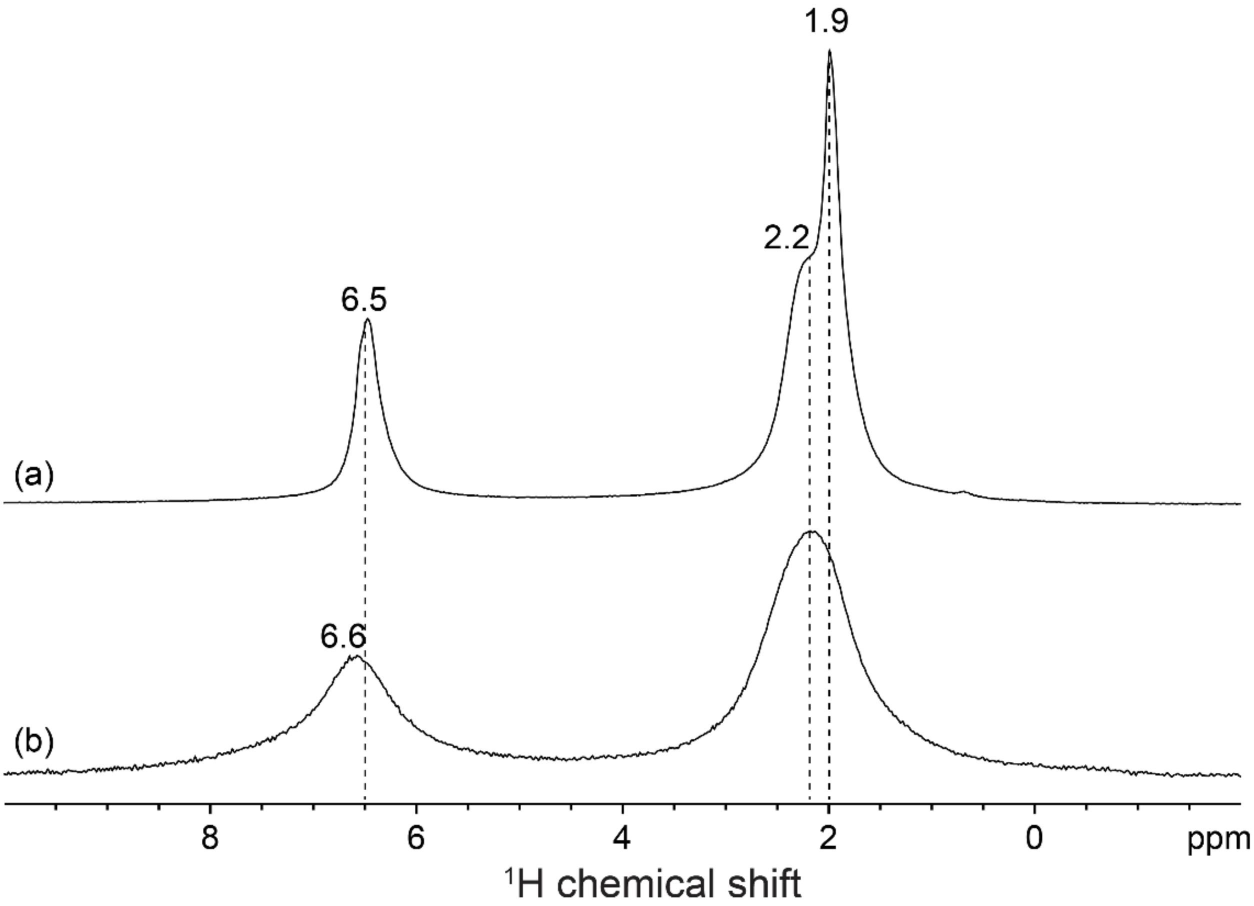

Figure S4: Solid-state ^{1}H echo MAS NMR spectrum of $(Mes)_2Al(OSi\equiv)$ (a) before and (b) after thermal treatment at 400 °C, acquired at 16.4 T, 10 kHz MAS, 295 K, and with an echo delay of one rotor period. The ^{1}H signals at 1.9-2.2 ppm are assigned to mesityl methyl ^{1}H species, while those at 6.5-6.6 ppm are assigned to mesityl aromatic ^{1}H species. The slight shift and broadening of the ^{1}H signals after thermal treatment indicates a broader distribution of surface-bound organic species, consistent with the transfer of mesityl groups from surface Al to surface Si atoms.

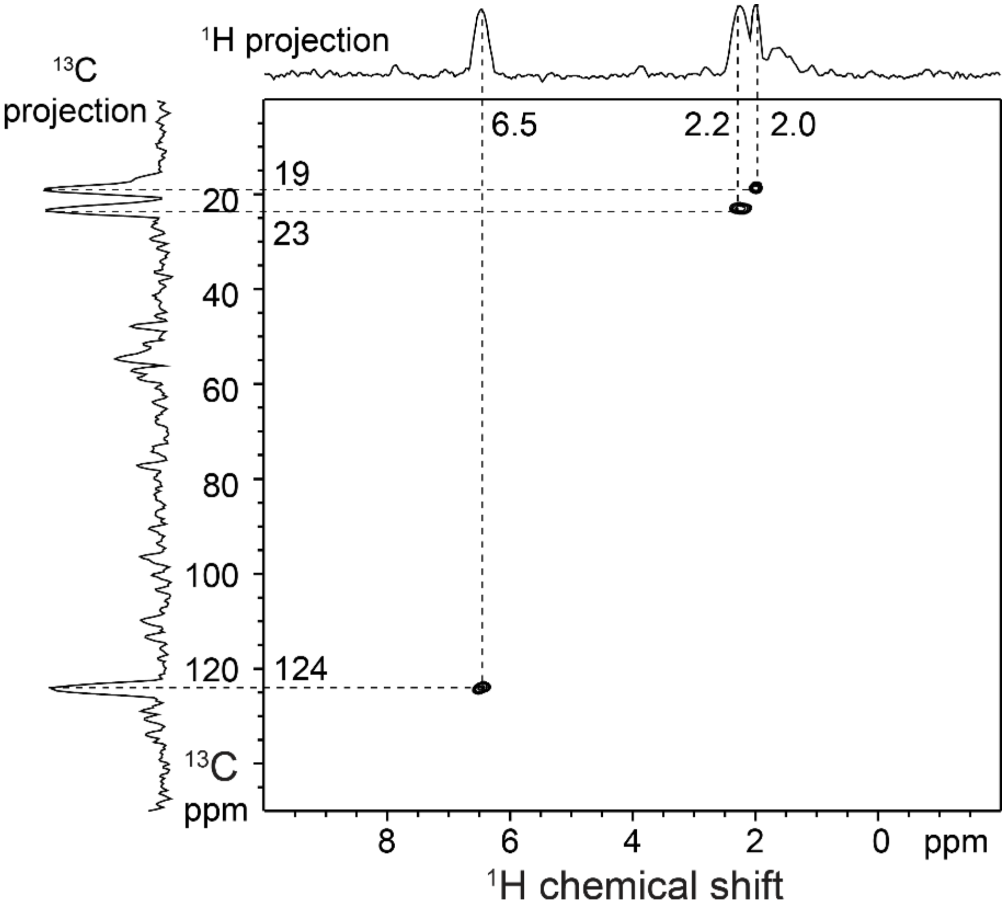

Figure S5: Solid-state 2D $^1H\{^{13}C\}$ heteronuclear multiple quantum correlation (HMQC) NMR spectrum of echo MAS NMR spectrum of$(Mes)_2Al(OSi\equiv)$ before temperature treatment acquired at 20.0 T, 50 kHz MAS, 295 K, and with 18 rotor periods (0.36 ms) of SR412 dipolar recoupling[S18] to reintroduce the ^1H-^{13}C dipole-dipole couplings. The correlated ^{13}C-^1H intensities at 2.2 ppm and 2.0 ppm in the ^1H dimension and 23 ppm and 19 ppm in the ^{13}C dimension are assigned to the two inequivalent methyl groups of Al-bound mesityl, which the correlation at 6.5 ppm in the ^1H dimension and 124 ppm in the ^{13}C dimension is assigned to the aromatic C-H moieties.

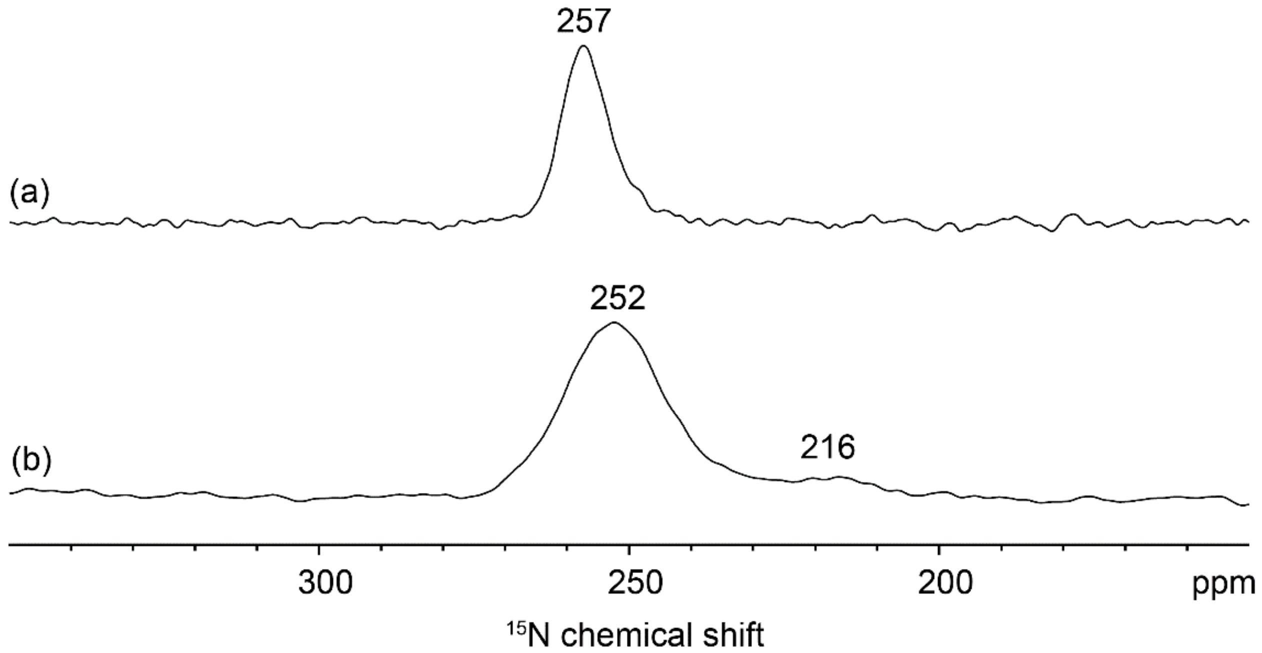

Figure S6: Solid-state $^{15}N\{^{1}H\}$ CP LTMAS NMR spectra of ^{15}N-pyridine adsorbed $(Mes)_2Al(OSi\equiv)$ (a) before and (b) after thermal treatment at 450 °C. Spectra were acquired at 14.1 T, 8 kHz MAS, 100 K and with ^{15}N-^{1}H contact times of 3 ms.

Characterization of M-Al@SiO$_2$

After contacting **Al@SiO$_2$** with (Cp^{ttt})$_2$MCl, the color of the material changed to a darker yellow. Liquid phase ^{1}H NMR spectrum of this process shows no release of Cp^{ttt}H, indicating a chemisorption of the organometallic precursor rather than a grafting via protonolysis. These data are corroborated by elemental analysis. Assuming the grafting and drying of the material does not influence the Al:Mes ration, elemental analysis of **Dy-Al@SiO$_2$** and **Y-Al@SiO$_2$** show a M:Cl ratio of 1:1 while the remaining C and H masses can be added up to 2 (Cp^{ttt})$^-$ ligands per metal center supporting the existence of an intact precursor interacting with the surface. This is further corroborated with solid-state ^{1}H magic-angle-spinning (MAS) NMR spectra of **Y-Al@SiO$_2$** which verify the presence of both –CH$_3$ and aromatic moieties on the surface (Fig. S8). Additionally, in the ^{13}C{^{1}H} CP-MAS spectra of **Y-Al@SiO$_2$** different ^{13}C NMR signals are detected at 32 and 28 ppm arising from distinct methyl groups of the Cp^{ttt} ligand, as well as a signal at 19 ppm from the methyl moieties of surface-bound mesityl groups. A ^{13}C{^{1}H} CP-MAS NMR spectrum acquired with sensitivity enhanced by dynamic nuclear polarization (DNP, Fig. S9)[S19,S20] additionally detect signals at 139 - 132 and 123 ppm from the aromatic carbon atoms of the Cp^{ttt} and mesityl groups, respectively.

The ^{29}Si{^{1}H} CP-CPMG NMR spectrum of **Y-Al@SiO$_2$** (Fig. S10c) shows signals from both the silicon oxide support and Si-Mes moieties, similar to (Mes)$_2$Al(OSi≡) after thermal treatment, indicating that the Si-Mes moieties are preserved after interaction with the Y precursor.

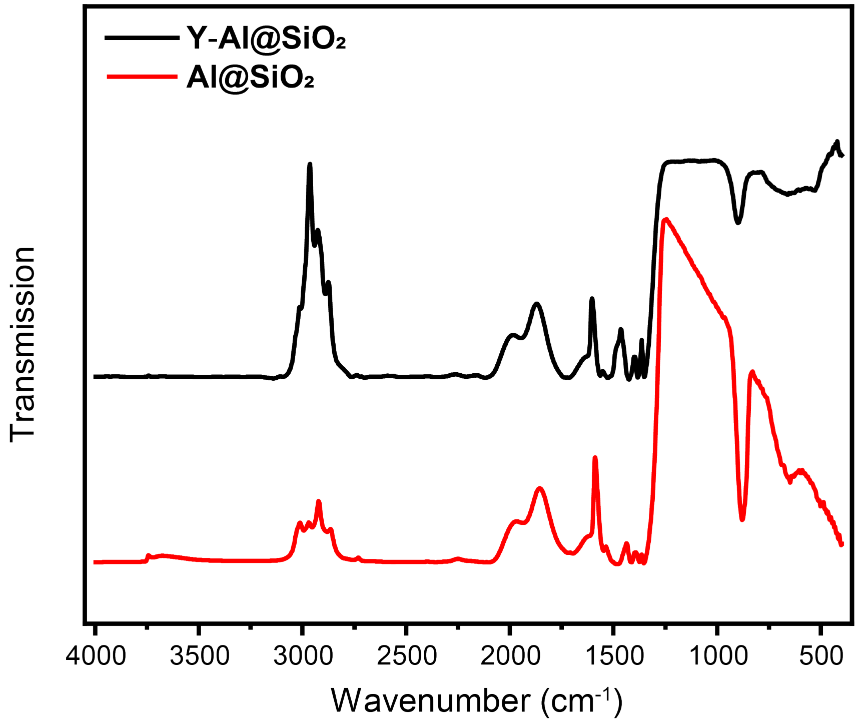

Figure S7: Transmission FT-IR of **Al@SiO$_2$** (red) compared to **Y-Al@SiO$_2$** (black).

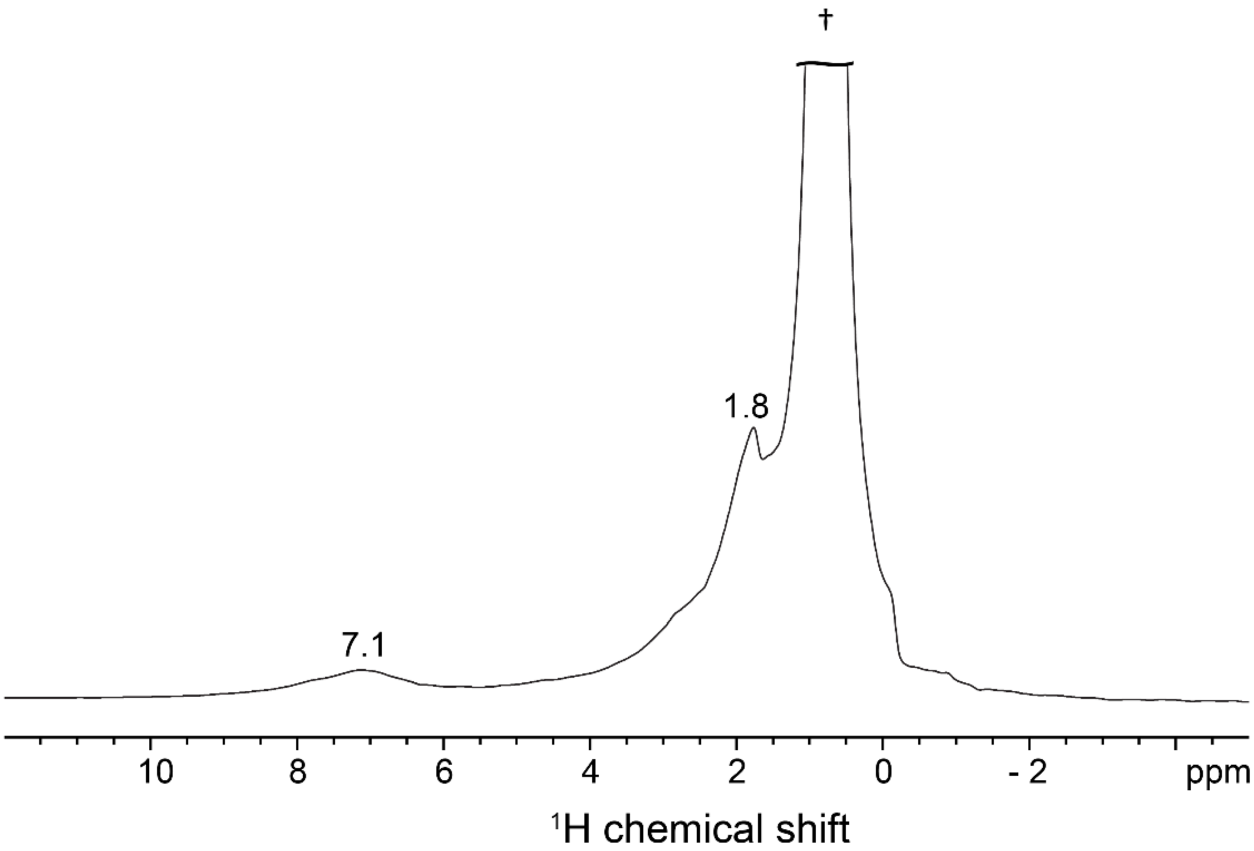

Figure S8: Solid-state ^{1}H echo MAS NMR spectrum of **Y-Al@SiO$_2$**, showing signals at 1.8 and 7.1 ppm arising from methyl and aromatic moieties, respectively. The spectrum was acquired at 16.4 T, 10 kHz MAS, 295 K, and with one rotor period echo delay. The **†** symbol indicates a background signal arising from the silicone plug.

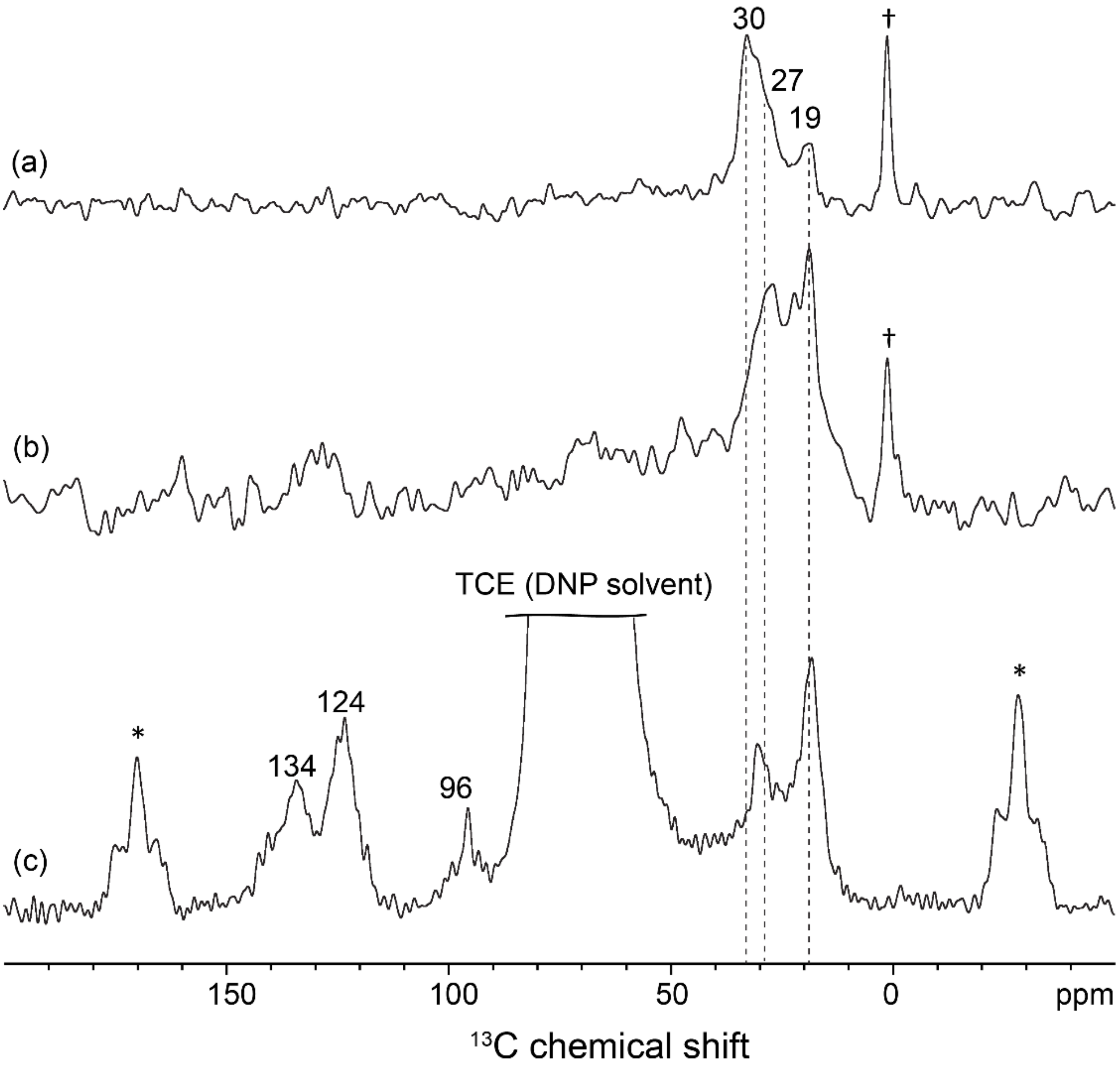

Figure S9: Solid-state $^{13}C\{^{1}H\}$ CPMAS NMR spectra of **Y-Al@SiO$_2$** acquired at 16.4 T, 10 kHz MAS, 295 K, and with ^{13}C-^{1}H contact times of (a) 2 ms and (b) 0.2 ms. The **†** symbol indicates a background signal arising from the silicone plug. The ^{13}C signals at 30 and 27 ppm arise from the distinct methyl groups of the $(Cp^{ttt})^{-}$ ligand, while the signal at 19 ppm is assigned to the methyl groups of surface mesitylene groups. (c) Solid-state $^{13}C\{^{1}H\}$ DNP-CPMAS NMR spectrum of **Y-Al@SiO$_2$** acquired at 9.4 T, 10 kHz MAS, 100 K, with a ^{13}C-^{1}H contact time of 2 ms, in the presence of 16 mM TEKPol biradical in 1,1,2,2-tetrachloroethane (DNP solvent), and under continuous microwave irradiation at 263 GHz. The DNP enhanced ^{13}C NMR spectrum shows the same signals from surface methyl groups, and additional reveals signals at 134 ppm and 124 ppm, which are assigned to the aromatic carbons of $(Cp^{ttt})^{-}$ and mesitylene, respectively. The weak signal at 96 ppm might arise from solvent decomposition or dilute ArO- residues formed on grafting of the molecular Y precursor. Asterisks indicate spinning sidebands.

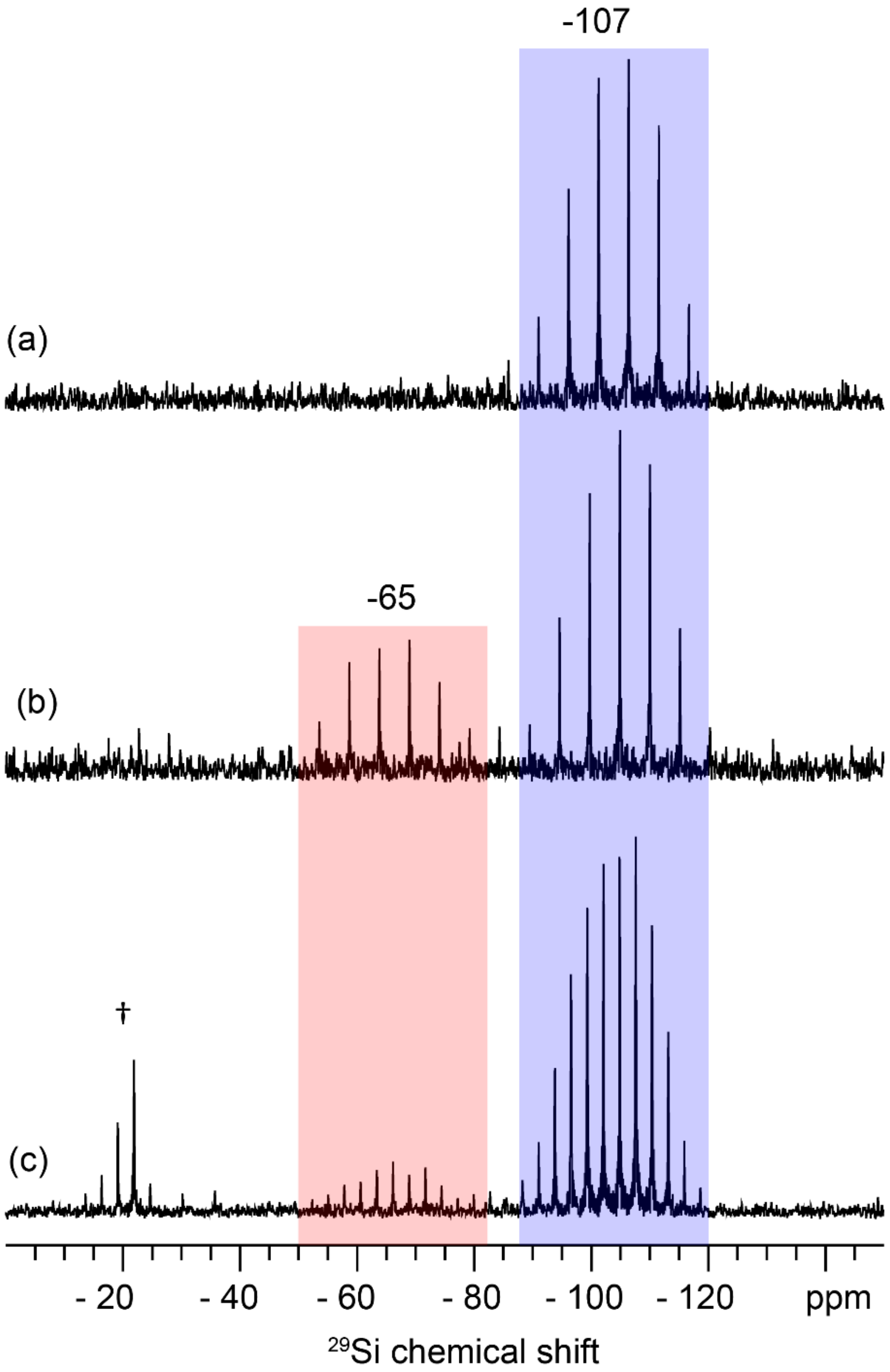

Figure S10: Solid-state $^{29}Si\{^{1}H\}$ CP-CPMG MAS NMR spectra of $(Mes)_2Al(OSi\equiv)$ (a) before and (b) after thermal treatment at 450 °C and (c) **Y-Al@SiO$_2$**. Blue and red shaded regions indicate signals arising from silica in all-oxygen tetrahedral environments and silicon with one carbon bond (e.g., Si-Mes), respectively. The **†** symbol indicates a background signal arising from the silicone plug. Spectra were acquired at 16.4 T, 10 kHz MAS, 298 K, and with ^{29}Si-^{1}H contact times of 8 ms.

Magnetism

The dynamic magnetic susceptibility (AC) measurements were performed on Quantum Design MPMS-XL SQUID magnetometer in the frequency range between 0.01-1000 Hz while the data points at 50, 100 and 250 Hz were omitted due to interference of harmonic waves of the instrument. The static susceptibility (DC) measurements of the sample was performed with a Quantum Design MPMS-XL SQUID magnetometer. In order to prevent any saturation effects during the measurement, a magnetic field of 0.2 kOe, 2 kOe and 10 kOe was used in the temperature ranges of 2-20 K, 20-80 K and 80-300 K, respectively. Due to the air sensitivity of **Dy-Al@SiO$_2$**, it was mixed with eicosane and the mixture was placed at the bottom of an EPR tube (4mm diameter) and attached to the high vacuum line (10^{-5} mbar). The bottom of the tube was warmed at 40 °C to melt eicosane, which upon cooling formed a solid matrix adhering to the walls of the tube and the tube was sealed under high vacuum. The prepared sample contained 22.9 mg of **Dy-Al@SiO$_2$** (containing 0.26 mg Dy(III)) and 32.4 mg icosane. The measurements were all corrected for the intrinsic diamagnetic contribution as calculated with Pascal's constants[S21,S22] and also for the diamagnetic contribution of the eicosane.

In- and out-of-phase susceptibility data (χ' and χ") obtained in the AC measurements (in the 2-49 K temperature range) were fitted using the extended Debey model with shared parameter and the best fit parameter are shown in Table S1. To gain access to the relaxation parameters of **Dy-Al@SiO$_2$**, the specific relaxation times at each temperature were fitted to Eq. S1 and the results are shown in Table S2. Using a two component fit (Raman and Orbach processes) leads to a good representation of the measured data (see Fig. 4). As observed by the butterfly shape hysteresis loop and calculations, the relaxation of the magnetic moment also has a contribution of QTM. Fitting the dynamic data to a three component fit including QTM, Raman and Orbach processes leads to extremely high values for QTM which are scientifically insignificant for the magnetic relaxation (Table S2) and thus only two components were considered. In both fits the exponent of the power-law for the Raman

mechanism has a similar value which is lower than normally expected for SMMs. Nevertheless, Goodwin *et al.*[S3] found similar results for their investigations of chloride abstracted compound $[(Cp^{ttt})_2Dy]^+$ and further, values of n in a similar magnitude are reported for several other compounds with a similar architecture $(Cp^R)_2Ln$.[S23,S24]

$$\tau^{-1} = \tau_{QTM}^{-1} + \tau_0^{-1} e^{-\frac{U_{eff}}{kT}} + CT^n \qquad \text{(Eq. S1)}$$

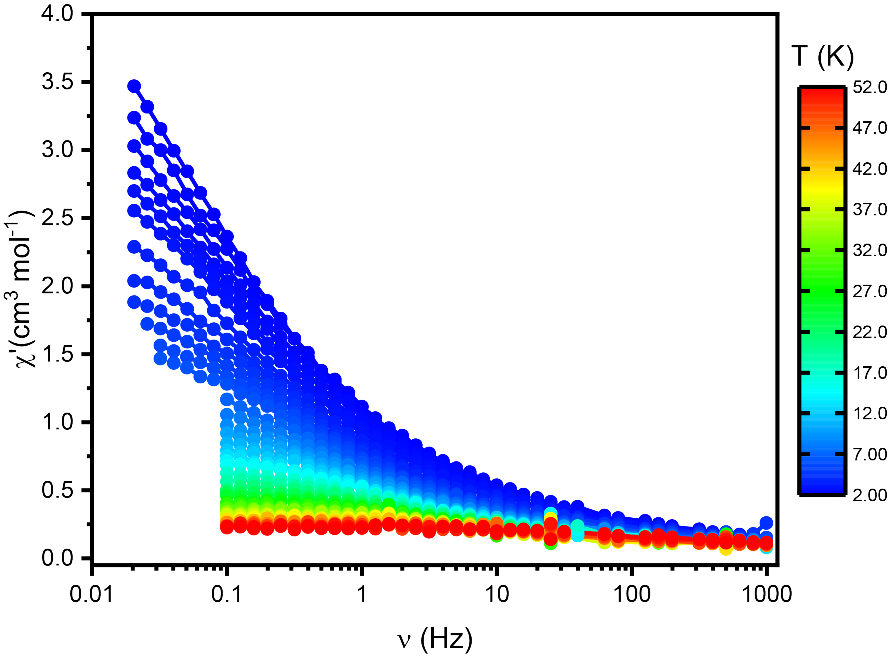

Figure S11: Frequency dependence of the in-phase component (χ') of the AC susceptibility of **Dy-Al@SiO$_2$** measured in zero external DC field between 2 and 51 K using a 3 Oe amplitude.

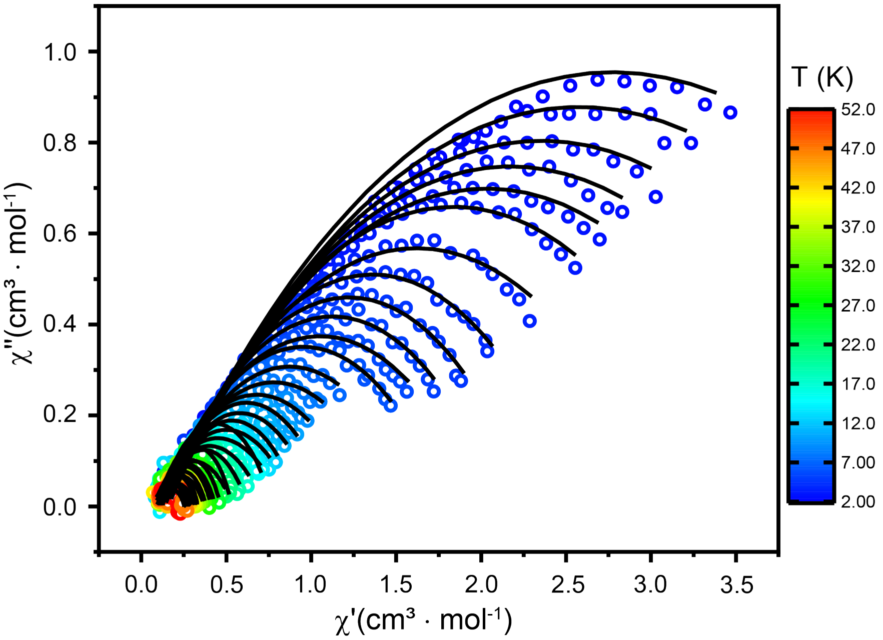

Figure S12: Variations of χ" as function of χ' (circles) for **Dy-Al@SiO$_2$** in zero external DC field. Circles represent experimental data points and lines represent the best fitted curves with extended Debye model.

Table S1: Best fitted parameter with the extended Debye model for **Dy-Al@SiO$_2$** at zero external DC field.

T (K)	χ_T ($cm^3 \cdot mol^{-1}$)	χ_S ($cm^3 \cdot mol^{-1}$)	α	τ (s)	R^2
2	5.41058	0.1384	0.55769	3.06238	0.99755
2.2	5.0037	0.14966	0.55795	2.70995	0.99639
2.4	4.58153	0.12568	0.55905	2.3672	0.99703
2.6	4.2142	0.13387	0.55289	2.10708	0.99842
2.8	3.91185	0.15449	0.54649	1.91747	0.99254
3	3.52093	0.15213	0.52542	1.48071	0.99422
3.5	3.09277	0.14906	0.53121	1.2457	0.98968
4	2.55869	0.15889	0.4884	0.81486	0.99252
4.5	2.28414	0.16058	0.47998	0.66576	0.99662
5	2.10718	0.14438	0.4878	0.59258	0.99271
5.5	2.00701	0.09736	0.52437	0.53864	0.98923
6	1.79097	0.11478	0.49516	0.43144	0.99441
7	1.62341	0.12367	0.50479	0.40382	0.99596
8	1.44647	0.11583	0.50432	0.33318	0.99777
9	1.27907	0.07733	0.50613	0.23118	0.92713
10	1.11207	0.12657	0.4482	0.19676	0.97439
11	1.02681	0.13241	0.45189	0.18482	0.94278
12	0.9274	0.13882	0.43109	0.14941	0.87999
13	0.90356	0.09803	0.49621	0.1304	0.94991
14	0.76585	0.10918	0.35599	0.1001	0.81276
15	0.83181	0.06783	0.52372	0.09362	0.97156
17	0.71791	0.08605	0.49339	0.07209	0.92802
19	0.62442	0.11103	0.42548	0.06387	0.9827

21	0.53134	0.10395	0.29658	0.04933	0.80713
23	0.5378	0.07986	0.47964	0.04489	0.97051
25	0.466	0.12386	0.31558	0.04341	0.97261
27	0.43584	0.09541	0.36297	0.02908	0.92862
29	0.41036	0.10959	0.32634	0.03497	0.98069
31	0.38142	0.10734	0.34831	0.02896	0.98825
33	0.36967	0.10632	0.40948	0.02335	0.96415
35	0.33299	0.09672	0.29894	0.01699	0.9519
37	0.32095	0.10757	0.28824	0.01779	0.99083
39	0.31402	0.11375	0.41393	0.01114	0.88621
41	0.2917	0.09609	0.39029	0.0086	0.94217
43	0.27757	0.10132	0.28267	0.01047	0.97026
45	0.2568	0.09505	0.2225	0.00703	0.92404
47	0.25411	0.09948	0.41705	0.00328	0.93638
49	0.24628	0.08485	0.48786	0.00198	0.96404

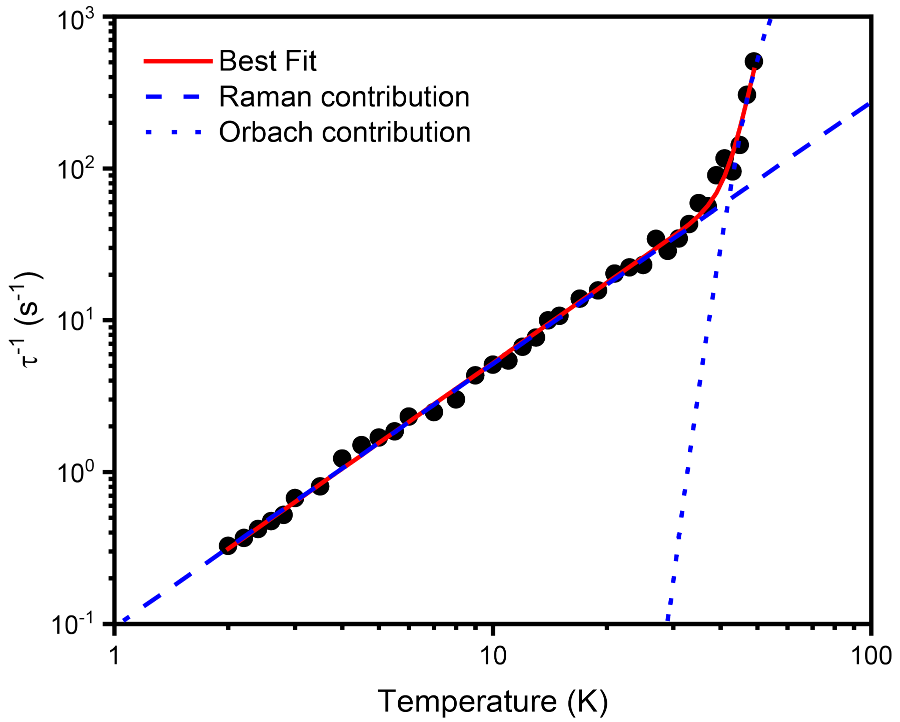

Figure S13: Temperature dependence of the relaxation time between 2 and 49 K where the red line is the best fit, blue dashed line considers only the Raman contribution and the blue dotted line considers only Orbach contribution. The fit parameters are shown in table S2.

Table S2: Parameters for the relaxation processes in **Dy-Al@SiO$_2$** obtained from fitting of AC measurement data using Equation Eq. S1, with the three terms describing quantum tunneling of magnetization (QTM), Orbach and Raman relaxation processes, respectively. Best fit parameters include a) only Orbach and Raman processes, b) QTM, Orbach and Raman relaxation.

	τ_{QTM} (s)	τ_0 (s)	U_{eff} (cm^{-1})	C ($s^{-1}\cdot K^{-n}$)	n
a)	-	$(5.24\pm9.87)\cdot10^{-9}$	448.74 ± 61.35	$(9.29\pm0.57)\cdot10^{-2}$	1.75 ± 0.03
b)	$(1.60\pm\ 0.00)\cdot10^{20}$	$(8.96\pm0.89)\cdot10^{-9}$	431.34 ± 59.16	$(9.34\pm2.33)\cdot10^{-2}$	1.75 ± 0.13

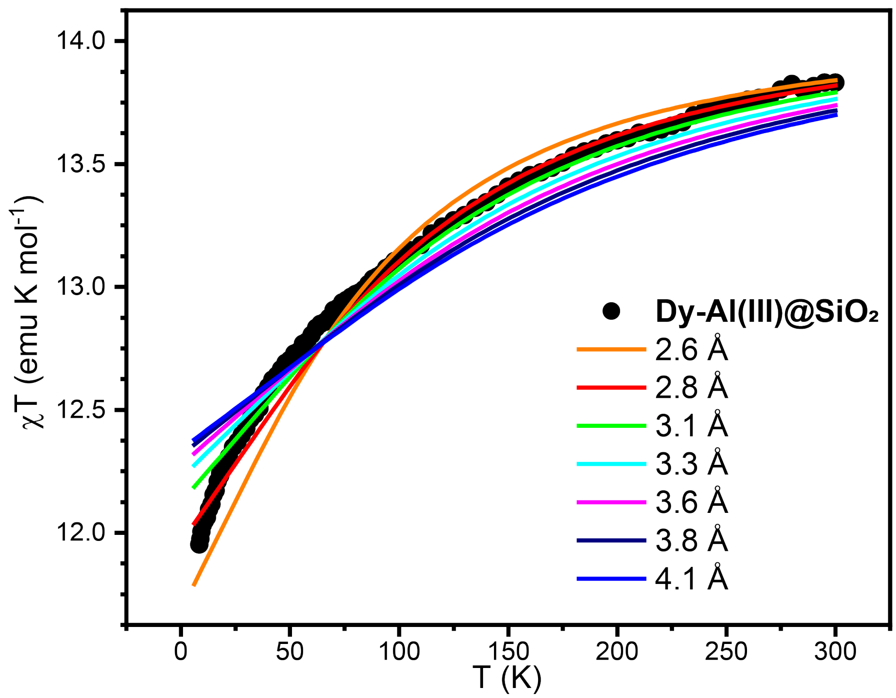

Figure S14: Temperature dependence of χT curve (black dots) of **Dy-Al@SiO$_2$** measured between 8.5 and 300 K compared with the calculated data for a Dy—Cl distance between 2.6 Å and 4.1 Å. Measured data were rescaled by 1.09 to match the high field value of pure dysprosium (5 Nβ per dysprosium according to ± 15/2 KD ground state).

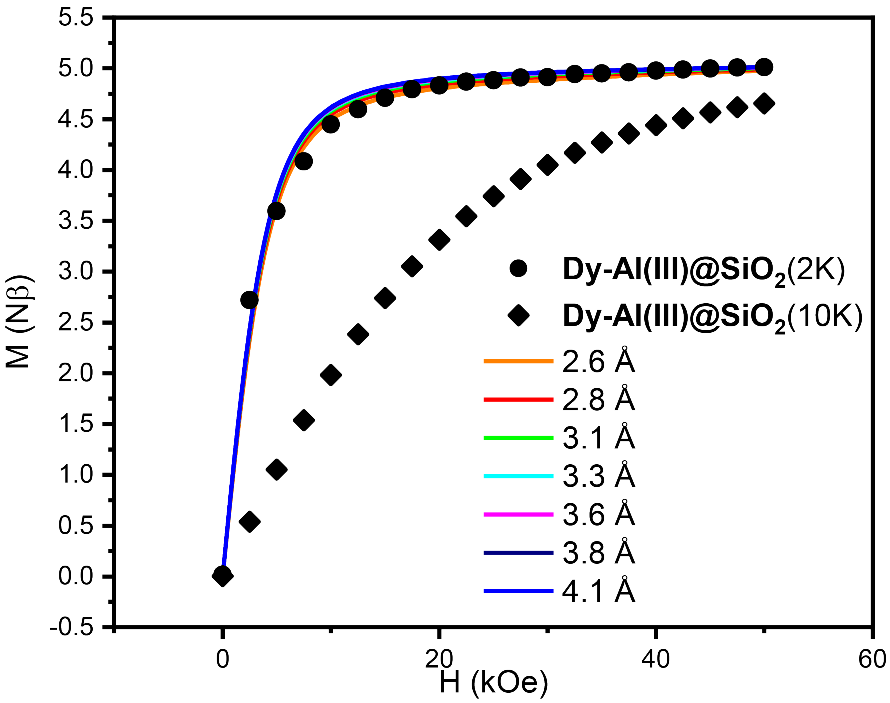

Figure S15: Magnetization as a function of the applied magnetic field at 2 K (black dots) of **Dy-Al@SiO$_2$** compared with the calculated data for a Dy—Cl distance between 2.6 Å and 4.1 Å. Measured data were rescaled by 1.09 to match the high field value of pure dysprosium (5 Nβ per dysprosium according to ± 15/2 KD ground state).

Computational methods

The coordinates of the molecular precursor $(Cp^{ttt})_2YCl$ were isolated from the published XRD structure[S3] and the geometry further optimized using the Gaussian 09 (revision d1) program suite.[S25] For all density functional calculations (DFT) the B3LYP[S26] functional was used in combination with the 6-31g(d)[S27] basis set for C, H and Cl while the LanL2DZ basis set and corresponding pseudopotential[S28] was used for Y. To simulate the abstraction of the Cl from Y the Y–Cl bond length was step-wise increased by 0.25 Å and the geometry optimized with the scan function implemented in the Gaussian program suite. The bond length was scanned between 2.6 and 4.1 Å.

Calculation of the electronic structure and magnetic properties of the models were performed with State-Averaged Complete Active Space Self-Consistent Field approach with Restricted Active Space State Interaction method (SA-CASSCF/RASSI-SO) included in the Open-Molcas 19.11 version.[S29] In the calculated models Y was replaced by Dy to represent the paramagnetic congener of these compounds. The performed multi-configurational approach used the Douglas-Kroll Hamiltonian to treat the relativistic effects in two steps. In the basis set generation scalar terms are included and are further used in the determination of the CASSCF wave functions and energies.[S30] The calculated CASSCF wave functions are mixed within the RASSI-SO method to account for spin-orbit coupling.[S31,S32] The active space was chosen to span the seven 4f orbitals populated by nine 4f electrons. In described active space, state-averaged CASSCF calculations were performed on all 21 sextets, all 224 quartets and all 490 doublets. These excited states were mixed using the RASSI-SO module which includes all 21 sextets, 128 quartets and 130 doublets. The atomic natural (ANO_RCC) basis set with the following contractions was employed for the description of the different atoms: [8s7p5d4f2g1h] for Dy; [5s4p3d1f] for Cl; [4s3p2d1f] for C and [2s1p] for H.[S33,S34] To speed up the calculation and save disc space the Cholesky decomposition of the bielectronic integrals was employed.[S35] In the final step, the magnetic properties and the g-tensors of the ground state multiplet was computed using the SINGLE_ANISO routine.[S36]

Visualization of the blocking barrier for each model was done with CAMMEL. The CAMMEL code is available under GNU General Public License v3.0 and can be downloaded at https://github.com/rmarchal1/CAMMEL.

Table S3: Computed energy levels (the ground state is set at zero), composition of the g-tensor and contributions to the wave function for each M_J state of the ground-state multiplet for the models of $(Cp^{ttt})_2DyCl$ with Dy–Cl bond distances of 2.6 and 2.8 Å. KD stands for Kramers doublet.

KD	E (cm^{-1})	g_x	g_y	g_z	wave function composition*
**2.6 Å**					
1	0.0	0.0	0.1	19.3	91.0 ± —15/2>
2	179.9	1.4	3.6	13.8	64.6 ± —13/2>+ 20.1 ± —9/2>
3	263.2	2.1	4.8	10.6	24.0 ± —3/2>+ 21.0 ± —7/2>+ 18.6 ± —1/2>+ 14.0 ± —13/2>+ 12.0 ± —11/2>
4	373.2	2.7	3.9	8.6	40.3 ± —11/2>+ 17.8 ± —5/2>+ 10.8 ± —1/2>+ 10.2 ± —7/2>
5	506.6	0.9	1.0	11.9	32.3 ± —9/2>+ 25.1 ± —11/2>+ 16.9 ± —3/2>
6	661.6	0.0	0.0	14.7	31.6 ± —7/2>+ 30.0 ± —9/2>+ 13.3 ± —1/2>+ 12.1 ± —11/2>
7	857.5	0.0	0.0	17.2	35.0 ± —5/2>+ 25.8 ± —7/2>+ 23.2 ± —3/2>+ 10.0 ± —9/2>
8	1120.1	0.0	0.0	19.8	46.5 ± —1/2>+ 32.5 ± —3/2>+ 15.4 ± —5/2>
**2.8 Å**					
1	0.0	0.0	0.0	19.6	94.3 ± —15/2>
2	227.9	0.3	0.4	16.1	83.9 ± —13/2>+ 13.4 ± —9/2>
3	375.6	8.6	8.2	3.4	40.1 ± —11/2>+ 25.1 ± —7/2>+ 15.0 ± —3/2>
4	454.2	0.3	3.4	8.2	27.3 ± —11/2>+ 24.5 ± —5/2>+ 18.2 ± —1/2>+ 14.8 ± —9/2>
5	571.0	1.2	1.4	11.6	37.3 ± —9/2>+ 19.6 ± —3/2>+ 17.1 ± —11/2>+ 10.9 ± —7/2>
6	713.8	0.2	0.2	14.5	35.5 ± —7/2>+ 25.1 ± —9/2>+ 15.0 ± —1/2>+ 13.7 ± —5/2>
7	901.5	0.0	0.0	17.1	36.7 ± —5/2>+ 26.5 ± —3/2>+ 23.3 ± —7/2>
8	1162.9	0.0	0.0	19.7	48.2 ± —1/2>+ 32.6 ± —3/2>+ 14.5 ± —5/2>

* Contributions < 10% are omitted.

Table S4: Computed energy levels (the ground state is set at zero), composition of the g-tensor and contributions to the wave function for each M_J state of the ground-state multiplet for the models of $(Cp^{ttt})_2DyCl$ with Dy–Cl bond distances of 3.1 and 3.3 Å. KD stands for Kramers doublet.

KD	E (cm^{-1})	g_x	g_y	g_z	wave function composition*
**3.1 Å**					
1	0.0	0.0	0.0	19.7	96.5 ± —15/2>
2	266.7	0.0	0.1	16.6	91.1 ± —13/2>
3	455.8	1.1	1.8	13.0	74.5 ± —11/2>+ 16.6 ± —7/2>
4	557.8	5.6	6.1	8.0	36.4 ± —9/2>+ 26.9 ± —5/2>+ 16.1 ± —1/2>
5	645.8	0.3	1.2	10.9	31.1 ± —9/2>+ 24.4 ± —3/2>+ 21.1 ± —7/2>+ 11.0 ± —11/2>
6	772.9	0.3	0.4	14.1	37.8 ± —7/2>+ 20.6 ± —5/2>+ 18.3 ± —9/2>+ 17.5 ± —1/2>
7	947.6	0.0	0.1	16.9	38.0 ± —5/2>+ 31.0 ± —3/2>+ 19.7 ± —7/2>
8	1195.6	0.0	0.0	19.7	50.6 ± —1/2>+ 32.7 ± —3/2>+ 13.1 ± —5/2>
**3.3 Å**					
1	0.0	0.0	0.0	19.8	97.8 ± —15/2>
2	301.4	0.0	0.0	16.8	94.9 ± —13/2>
3	511.3	0.2	0.3	14.0	88.0 ± —11/2>
4	645.2	2.9	4.0	9.8	68.1 ± —9/2>+ 18.2 ± —5/2>
5	728.8	3.9	5.9	8.5	39.0 ± —7/2>+ 27.3 ± —3/2>+ 13.1 ± —1/2>+ 11.5 ± —9/2>
6	834.7	0.2	0.5	13.4	33.8 ± —7/2>+ 30.2 ± —5/2>+ 20.9 ± —1/2>+ 12.0 ± —9/2>
7	994.0	0.0	0.1	16.5	38.0 ± —5/2>+ 36.5 ± —3/2>+ 15.4 ± —7/2>
8	1223.2	0.0	0.0	19.5	53.6 ± —1/2>+ 32.5 ± —3/2>+ 11.5 ± —5/2>

* Contributions < 10% are omitted.

Table S5: Computed energy levels (the ground state is set at zero), composition of the g-tensor and contributions to the wave function for each M_J state of the ground-state multiplet for the models of $(Cp^{ttt})_2DyCl$ with Dy–Cl bond distances of 3.6 and 3.8 Å. KD stands for Kramers doublet.

KD	E (cm^{-1})	g_x	g_y	g_z	wave function composition*
					**3.6 Å**
1	0.0	0.0	0.0	19.8	98.5 ± 15/2>
2	328.9	0.0	0.0	16.9	96.8 ± 13/2>
3	551.4	0.1	0.1	14.2	93.4 ± 11/2>
4	703.5	0.9	1.1	11.3	85.9 ± 9/2>
5	809.3	5.3	5.8	6.7	62.8 ± 7/2>+ 20.8 ± 3/2>
6	900.4	2.0	3.4	11.6	43.5 ± 5/2>+ 25.3 ± 1/2>+ 19.6 ± 7/2>
7	1039.6	0.1	0.2	16.0	43.2 ± 3/2>+ 35.0 ± 5/2>+ 11.1 ± 7/2>
8	1250.8	0.0	0.0	19.4	56.6 ± 1/2>+ 31.9 ± 3/2>
					**3.8 Å**
1	0.0	0.0	0.0	19.8	99.1 ± 15/2>
2	352.2	0.0	0.0	17.0	98.1 ± 13/2>
3	584.1	0.0	0.0	14.3	96.6 ± 11/2>
4	745.5	0.3	0.3	11.6	93.4 ± 9/2>
5	869.2	2.9	3.3	8.1	81.6 ± 7/2>+ 12.0 ± 3/2>
6	962.4	3.5	5.9	9.2	57.6 ± 5/2>+ 24.5 ± 1/2>
7	1082.0	0.5	1.0	15.2	50.7 ± 3/2>+ 29.2 ± 5/2>+ 11.9 ± 1/2>
8	1269.2	0.0	0.0	19.2	60.4 ± 1/2>+ 30.7 ± 3/2>

* Contributions < 10% are omitted.

Table S6: Computed energy levels (the ground state is set at zero), composition of the g-tensor and contributions to the wave function for each M_J state of the ground-state multiplet for the models of $(Cp^{ttt})_2DyCl$ with Dy–Cl bond distance of 4.1 Å. KD stands for Kramers doublet.

KD	E (cm^{-1})	g_x	g_y	g_z	wave function composition*
**4.1 Å**					
1	0.0	0.0	0.0	19.9	99.4 ± —15/2>
2	373.2	0.0	0.0	17.0	98.9 ± —13/2>
3	611.1	0.0	0.0	14.4	98.1 ± —11/2>
4	777.6	0.1	0.1	11.7	96.7 ± —9/2>
5	913.4	1.3	1.4	8.7	91.8 ± —7/2>
6	1020.1	6.7	6.2	4.7	71.9 ± —5/2>+ 18.6 ± —1/2>
7	1123.9	1.2	2.7	14.0	59.7 ± —3/2>+ 19.8 ± —5/2>+ 15.8 ± —1/2>
8	1288.3	0.1	0.2	19.0	64.8 ± —1/2>+ 28.7 ± —3/2>

* Contributions < 10% are omitted.

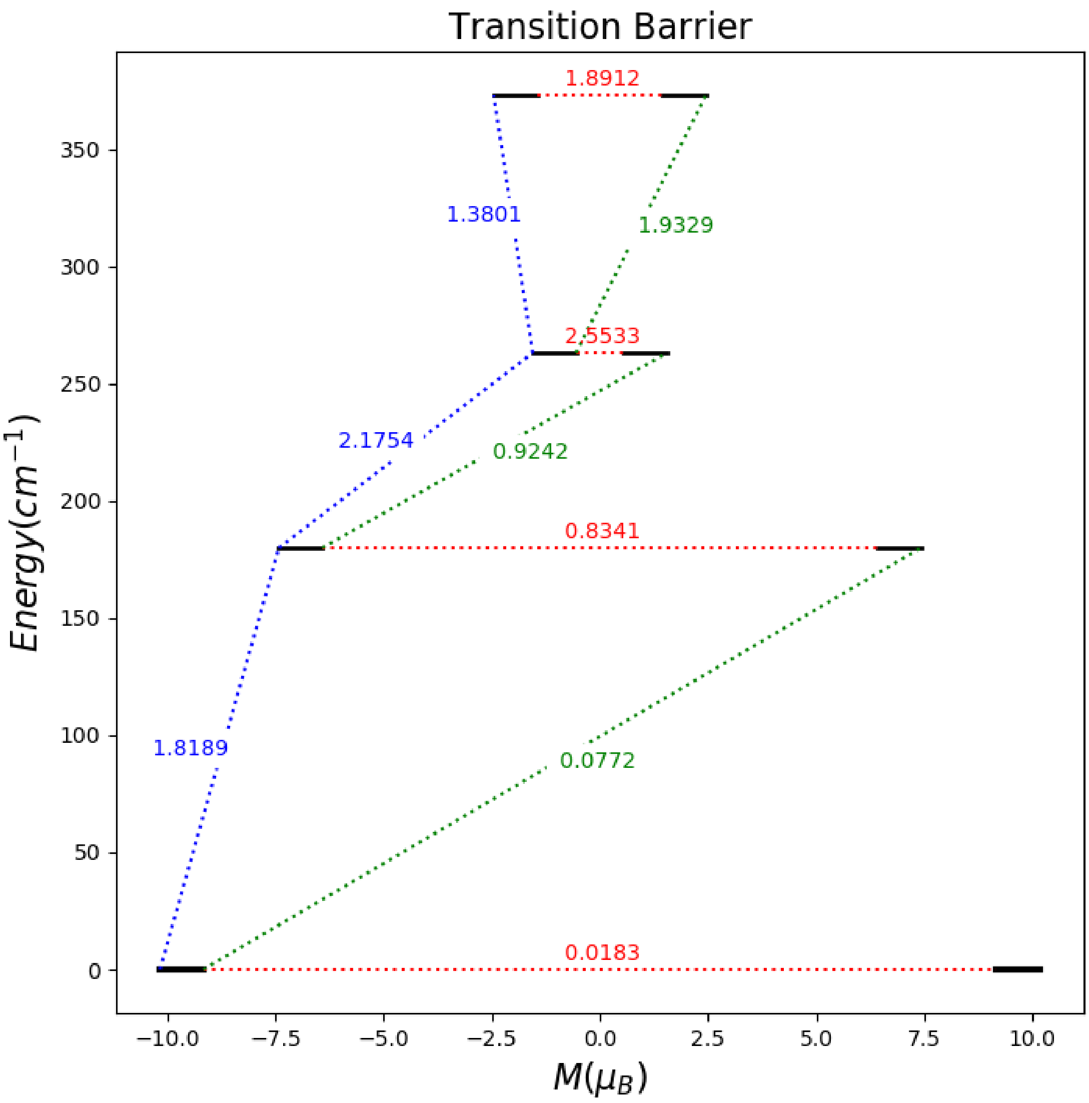

Figure S16: Computed magnetization blocking barriers the model of $(Cp^{ttt})_2DyCl$ with Dy–Cl bond distances of 2.6 Å. The four lowest Kramers doublets (thick black lines) are represented according to their magnetic moment along the main magnetic axis. The blue lines represent vertical excitations, the green dashed lines correspond to possible Orbach relaxation processes while the red lines correspond to QTM/TA-QTM processes. The values correspond to the mean value of the corresponding transversal matrix element of the transition magnetic moment.

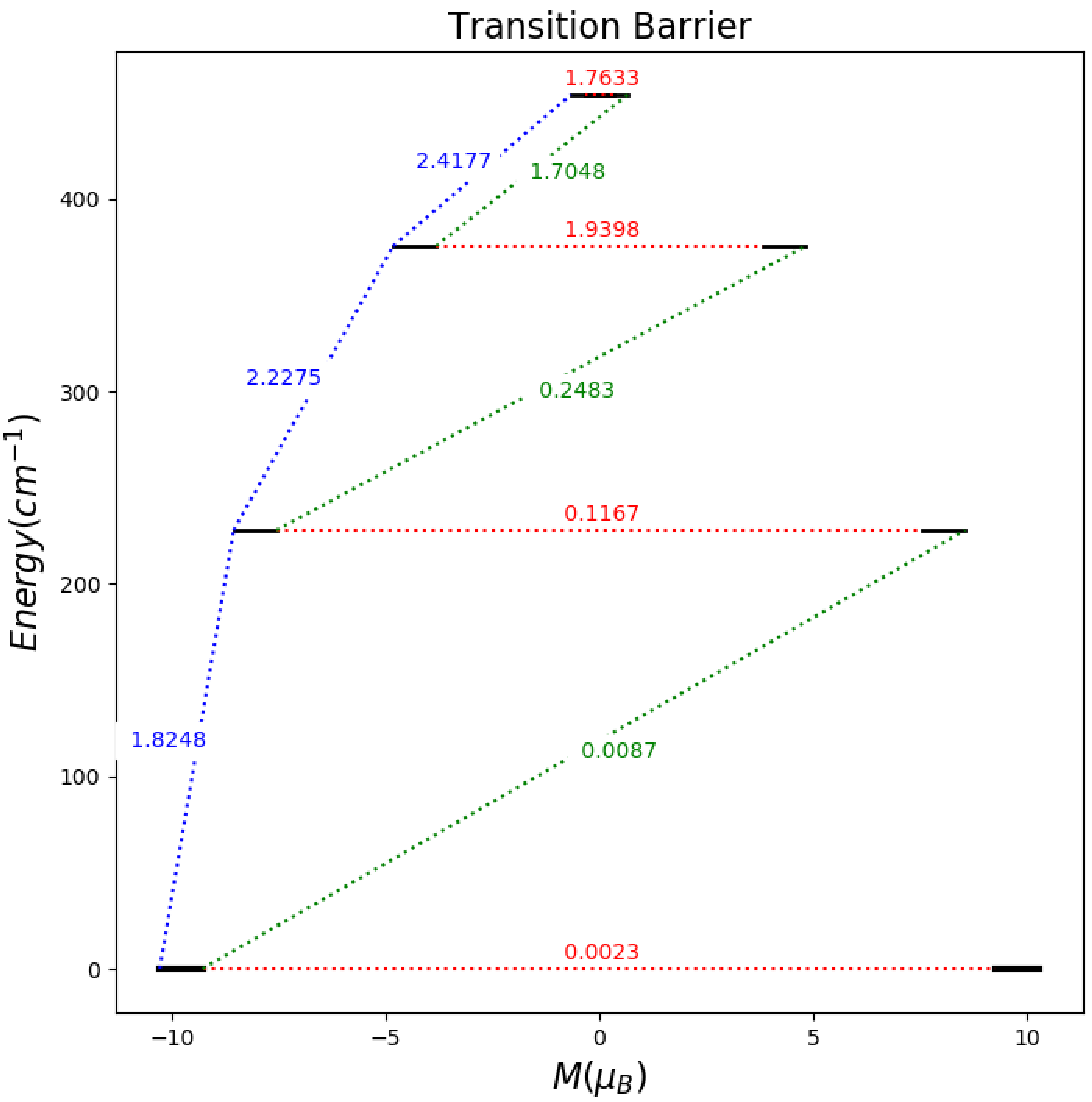

Figure S17: Computed magnetization blocking barriers the model of $(Cp^{ttt})_2DyCl$ with Dy–Cl bond distances of 2.8 Å. The four lowest Kramers doublets (thick black lines) are represented according to their magnetic moment along the main magnetic axis. The blue lines represent vertical excitations, the green dashed lines correspond to possible Orbach relaxation processes while the red lines correspond to QTM/TA-QTM processes. The values correspond to the mean value of the corresponding transversal matrix element of the transition magnetic moment.

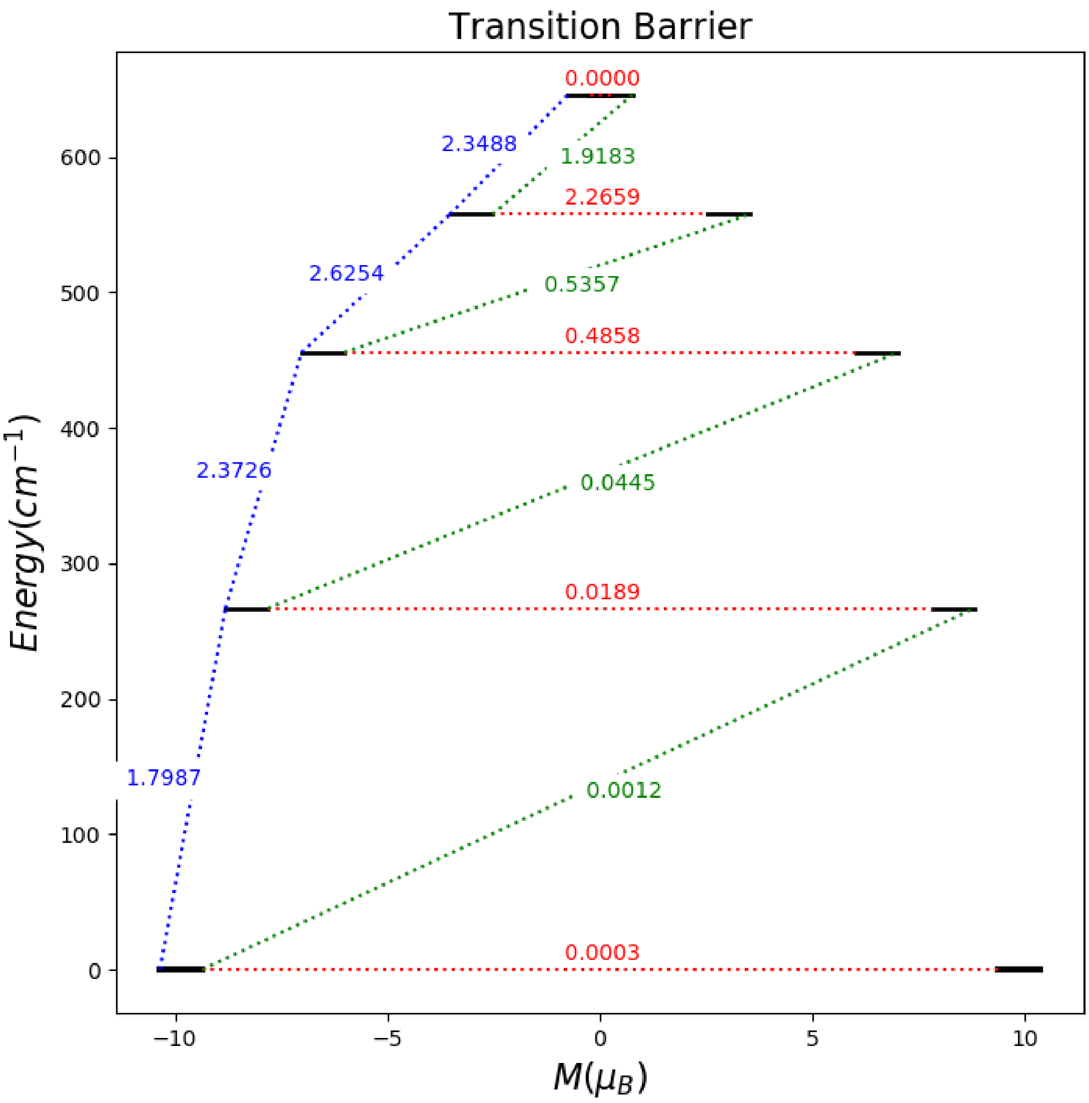

Figure S18: Computed magnetization blocking barriers the model of $(Cp^{ttt})_2DyCl$ with Dy–Cl bond distances of 3.1 Å. The four lowest Kramers doublets (thick black lines) are represented according to their magnetic moment along the main magnetic axis. The blue lines represent vertical excitations, the green dashed lines correspond to possible Orbach relaxation processes while the red lines correspond to QTM/TA-QTM processes. The values correspond to the mean value of the corresponding transversal matrix element of the transition magnetic moment.

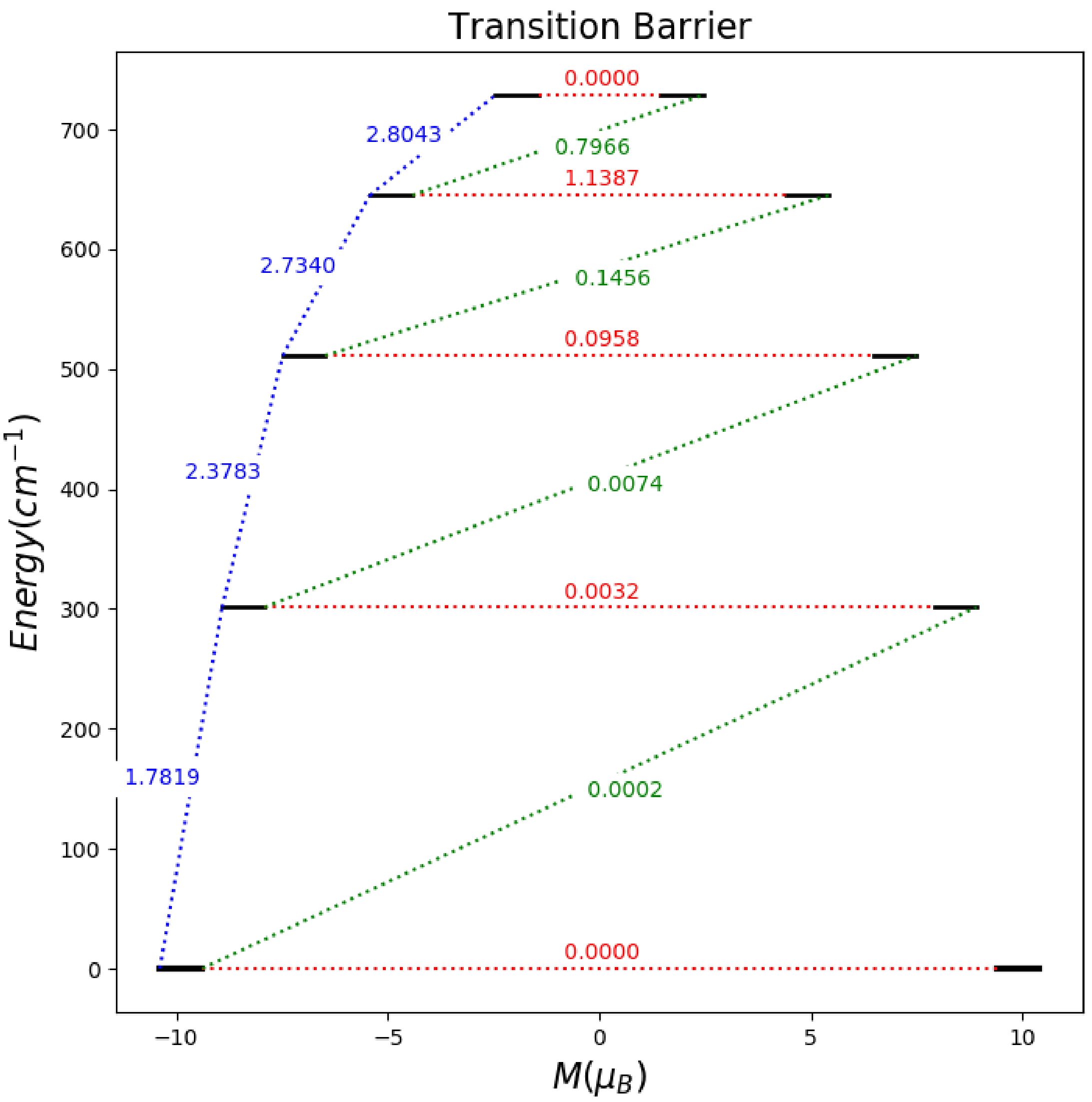

Figure S19: Computed magnetization blocking barriers the model of $(Cp^{ttt})_2DyCl$ with Dy–Cl bond distances of 3.3 Å. The four lowest Kramers doublets (thick black lines) are represented according to their magnetic moment along the main magnetic axis. The blue lines represent vertical excitations, the green dashed lines correspond to possible Orbach relaxation processes while the red lines correspond to QTM/TA-QTM processes. The values correspond to the mean value of the corresponding transversal matrix element of the transition magnetic moment.

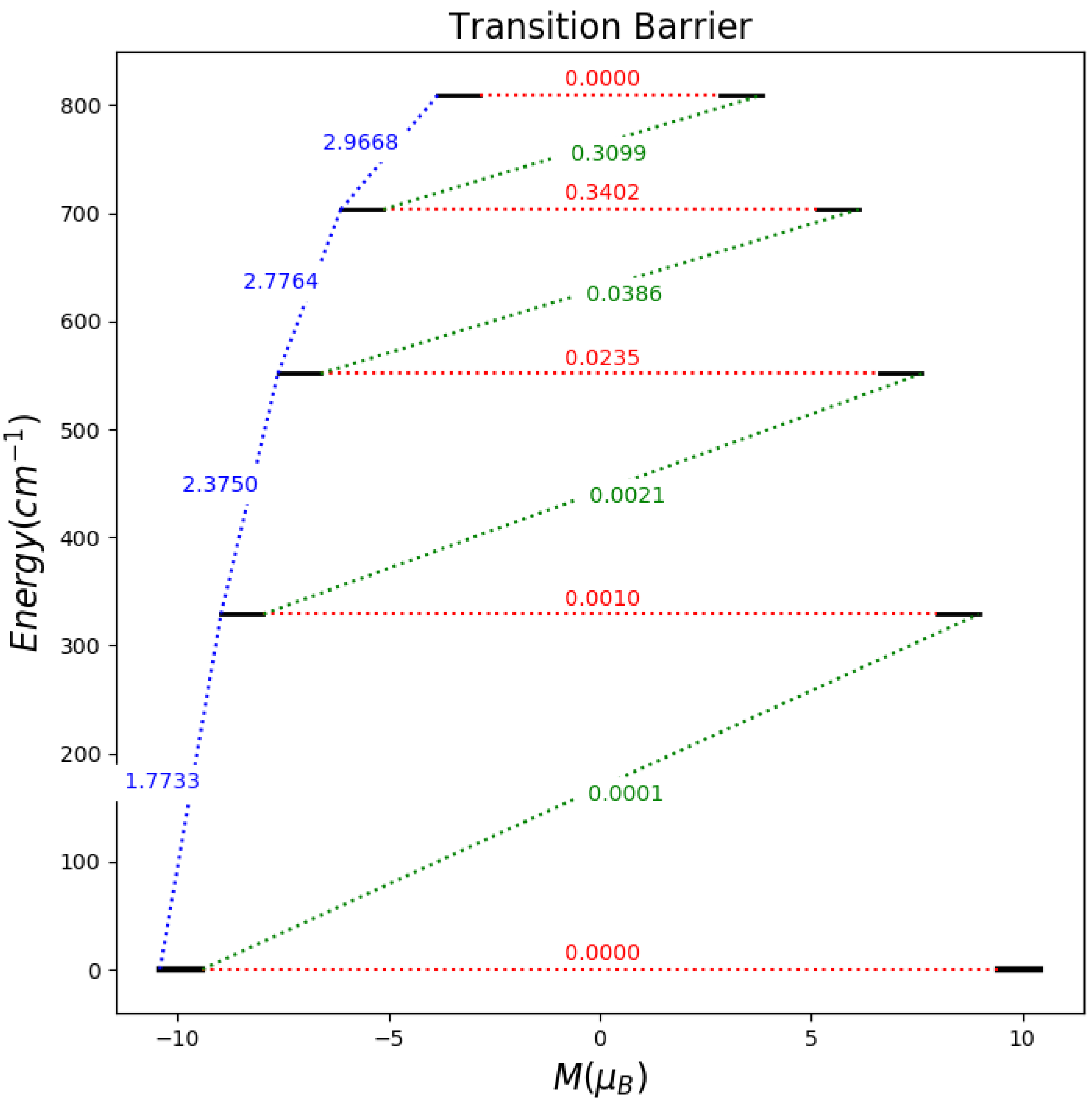

Figure S20: Computed magnetization blocking barriers the model of $(Cp^{ttt})_2DyCl$ with Dy–Cl bond distances of 3.6 Å. The four lowest Kramers doublets (thick black lines) are represented according to their magnetic moment along the main magnetic axis. The blue lines represent vertical excitations, the green dashed lines correspond to possible Orbach relaxation processes while the red lines correspond to QTM/TA-QTM processes. The values correspond to the mean value of the corresponding transversal matrix element of the transition magnetic moment.

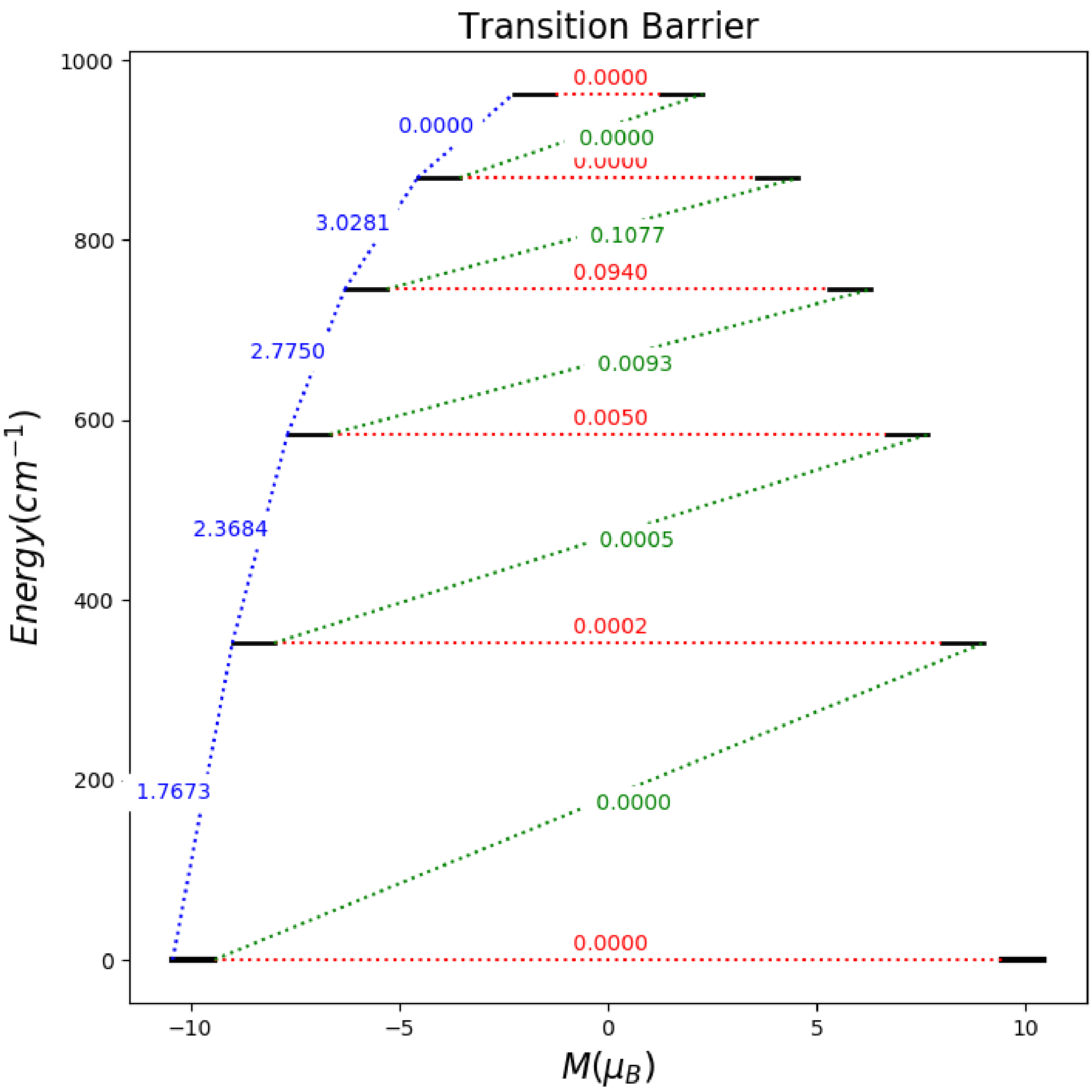

Figure S21: Computed magnetization blocking barriers the model of $(Cp^{ttt})_2DyCl$ with Dy–Cl bond distances of 3.8 Å. The four lowest Kramers doublets (thick black lines) are represented according to their magnetic moment along the main magnetic axis. The blue lines represent vertical excitations, the green dashed lines correspond to possible Orbach relaxation processes while the red lines correspond to QTM/TA-QTM processes. The values correspond to the mean value of the corresponding transversal matrix element of the transition magnetic moment.

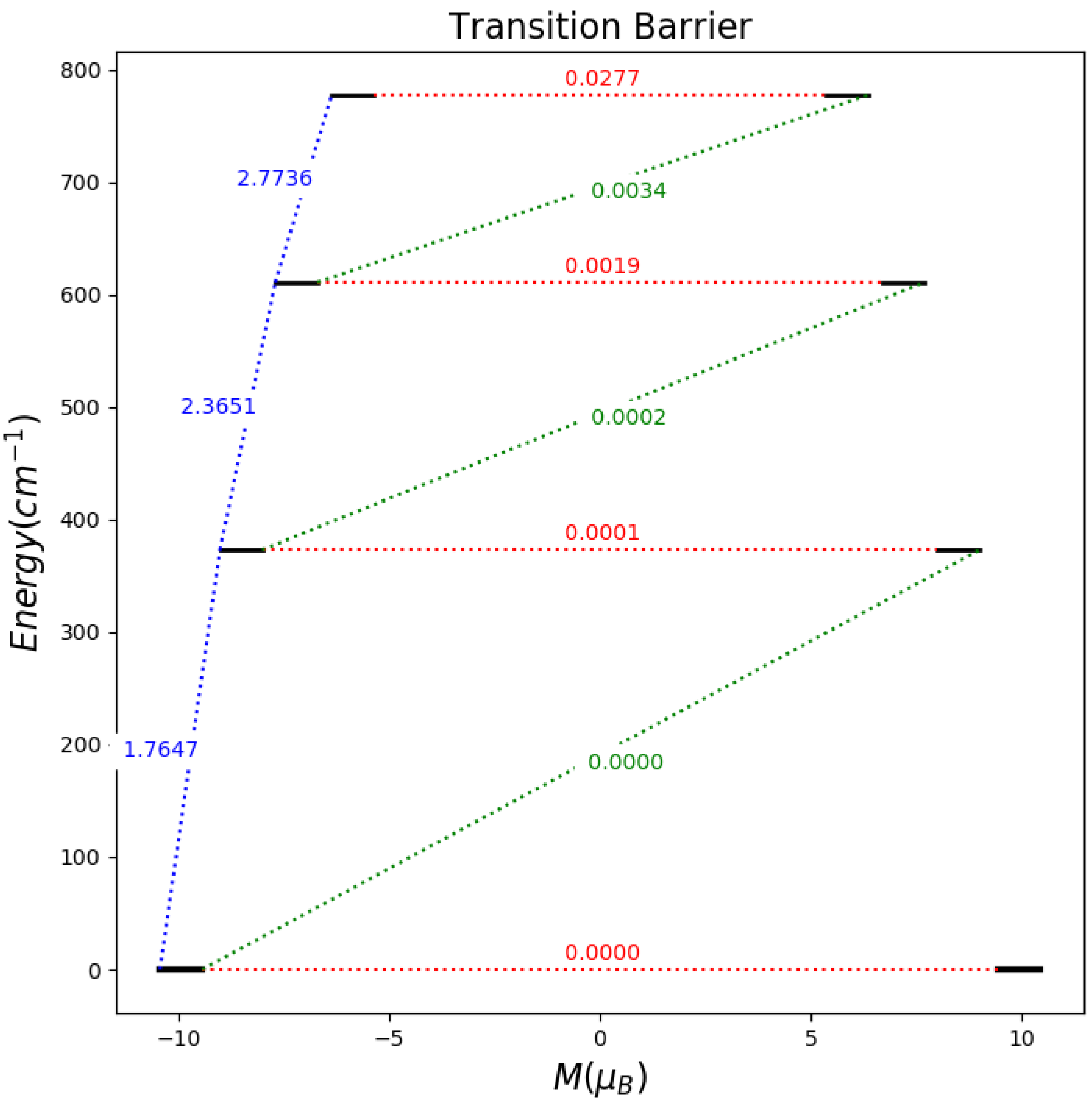

Figure S22: Computed magnetization blocking barriers the model of $(Cp^{ttt})_2DyCl$ with Dy–Cl bond distances of 4.1 Å. The four lowest Kramers doublets (thick black lines) are represented according to their magnetic moment along the main magnetic axis. The blue lines represent vertical excitations, the green dashed lines correspond to possible Orbach relaxation processes while the red lines correspond to QTM/TA-QTM processes. The values correspond to the mean value of the corresponding transversal matrix element of the transition magnetic moment.

Cartesian coordinates of computational models

2.6 Å				2.8 Å			
Atom	x	y	z	Atom	x	y	z
Dy	0.000095	-0.000437	-0.515010	Dy	-0.000242	0.000692	-0.476249
Cl	0.000285	-0.002458	-3.083588	Cl	0.000020	0.002867	-3.294827
C	-2.744243	-0.222713	-0.481305	C	-2.729150	-0.223591	-0.473277
C	-2.355403	-0.028741	0.913335	C	-2.351436	-0.030733000	0.924117
C	-1.608677	-1.199877	1.282246	C	-1.606330	-1.203224	1.297995
H	-1.248627	-1.402295	2.280224	H	-1.256491	-1.407923	2.299275
C	-1.537930	-2.113946	0.203901	C	-1.527921	-2.116078	0.219154
C	-2.200366	-1.479402	-0.877102	C	-2.177318	-1.477864	-0.868155
H	-2.308017	-1.908235	-1.861853	H	-2.272917	-1.902138	-1.856205
C	-3.678490	0.554480	-1.447321	C	-3.651434	0.556083	-1.448734
C	-5.119047	0.617136	-0.883155	C	-5.095266	0.629694	-0.894980
H	-5.202997	1.219934	0.022011	H	-5.183062	1.233357	0.009237
H	-5.487598	-0.388953	-0.652236	H	-5.473430	-0.373645	-0.667373
C	-3.791112	-0.181001	-2.806316	C	-3.757346	-0.182972	-2.806268
H	-4.237803	-1.175305	-2.695634	H	-4.208138	-1.175523	-2.695118
H	-4.444014	0.398487	-3.469137	H	-4.405311	0.396776	-3.473645
H	-2.820696	-0.281607	-3.300254	H	-2.783800	-0.285046	-3.293816
C	-2.888679	0.887442	2.047429	C	-2.891487	0.886100	2.054263
C	-3.202361	2.343827	1.650707	C	-3.197089	2.343448	1.654977
H	-4.017243	2.425721	0.931994	H	-4.001735	2.428276	0.925345
H	-3.504847	2.905687	2.542554	H	-3.509903	2.904746	2.543556
H	-2.326621	2.846994	1.228971	H	-2.314600	2.845144	1.245756
H	-3.966924	-0.769450	2.994270	H	-3.981924	-0.769809	2.989173

C	-1.882671	0.970864	3.222559	C	-1.894557	0.966208	3.237391
H	-0.920042	1.385522	2.908930	H	-0.928106	1.378423	2.931760
H	-2.289527	1.626243	4.001276	H	-2.305717	1.622897	4.012639
H	-1.698405	-0.000416	3.690044	H	-1.717026	-0.005523	3.706639
C	-1.116284	-3.585482	0.228727	C	-1.112376	-3.589191	0.245867
C	-0.558267	-3.990536	1.605302	C	-0.558421	-3.995638	1.623678
H	-0.268037	-5.047724	1.597520	H	-0.271184	-5.053577	1.616293
H	0.326049	-3.407643	1.881150	H	0.327060	-3.415412	1.901617
H	-1.308578	-3.857682	2.393014	H	-1.310017	-3.861130	2.409936
C	-2.374391	-4.446659	-0.046844	C	-2.375911	-4.442592	-0.030168
H	-2.793364	-4.236956	-1.036412	H	-2.791172	-4.232379	-1.021139
H	-2.123353	-5.514090	-0.004956	H	-2.131283	-5.511307	0.014621
H	-3.153870	-4.249161	0.697511	H	-3.155640	-4.238733	0.712240
C	-0.067573	-3.886160	-0.862257	C	-0.065111	-3.896295	-0.844429
H	-0.424528	-3.594935	-1.856429	H	-0.416613	-3.598924	-1.838714
H	0.870592	-3.351422	-0.673161	H	0.877783	-3.371655	-0.652226
H	0.169261	-4.957069	-0.891427	H	0.160984	-4.969365	-0.875831
H	-5.789935	1.057045	-1.631577	H	-5.756176	1.073843	-1.649332
C	-3.170643	1.976779	-1.776787	C	-3.127501	1.972503	-1.777721
H	-2.212246	1.928273	-2.306298	H	-2.165505	1.912460	-2.300363
H	-3.881474	2.483666	-2.441732	H	-3.827255	2.483335	-2.451200
H	-3.049674	2.602152	-0.892182	H	-3.009983	2.599700	-0.893677
C	-4.179361	0.235701	2.614045	C	-4.188375	0.236646	2.608935
H	-4.960882	0.146198	1.855736	H	-4.963831	0.151067	1.844006
H	-4.577616	0.835459	3.442654	H	-4.591421	0.836088	3.435324
C	2.355331	0.029634	0.913378	C	2.351332	0.029724	0.924095

C	2.744180	0.222818	-0.481358	C	2.729181	0.223376	-0.473132
C	2.199977	1.479112	-0.877972	C	2.177826	1.478102	-0.867233
H	2.307567	1.907378	-1.862973	H	2.273533	1.902926	-1.855029
C	1.537407	2.114209	0.202654	C	1.528596	2.115820	0.220434
C	1.608397	1.200854	1.281579	C	1.606577	1.202236	1.298693
H	1.248299	1.403799	2.279428	H	1.256804	1.406435	2.300100
C	2.888575	-0.886026	2.047882	C	2.891366	-0.887736	2.053751
C	1.882678	-0.968753	3.223168	C	1.894504	-0.968436	3.236894
H	1.698327	0.002823	3.689998	H	1.716963	0.003066	3.706613
H	0.920077	-1.383771	2.909940	H	0.928054	-1.380546	2.931132
H	2.289714	-1.623535	4.002286	H	2.305734	-1.625471	4.011814
C	3.201989	-2.342636	1.651769	C	3.196960	-2.344877	1.653697
H	3.504610	-2.904147	2.543785	H	3.509948	-2.906592	2.541953
H	2.326088	-2.845873	1.230450	H	2.314440	-2.846415	1.244373
H	4.016688	-2.424947	0.932890	H	4.001494	-2.429312	0.923893
C	4.179447	-0.234204	2.613967	C	4.188291	-0.238584	2.608694
H	3.967230	0.771195	2.993696	H	3.981873	0.767677	2.989450
H	4.577669	-0.833611	3.442837	H	4.591368	-0.838452	3.434761
H	4.960919	-0.145208	1.855525	H	4.963707	-0.152641	1.843767
C	3.678743	-0.554840	-1.446701	C	3.651248	-0.555890	-1.449118
C	3.171056	-1.977316	-1.775793	C	3.126961	-1.971973	-1.778860
H	3.049094	-2.602142	-0.890933	H	3.009784	-2.599738	-0.895177
H	2.213121	-1.928921	-2.306171	H	2.164746	-1.911472	-2.301042
H	3.882410	-2.484672	-2.439820	H	3.826342	-2.482468	-2.452988
C	5.119064	-0.617224	-0.881854	C	5.095104	-0.630180	-0.895534
H	5.487437	0.388955	-0.651058	H	5.473584	0.372940	-0.667489

H	5.202603	-1.219745	0.023546	H	5.182854	-1.234309	0.008374
H	5.790254	-1.057214	-1.629757	H	5.755837	-1.074168	-1.650187
C	3.791914	0.179985	-2.806016	C	3.757238	0.183996	-2.806180
H	4.238989	1.174140	-2.695677	H	4.208245	1.176383	-2.694423
H	4.444720	-0.400024	-3.468468	H	4.405045	-0.395469	-3.473959
H	2.821562	0.280655	-3.300133	H	2.783705	0.286660	-3.293617
C	1.115936	3.585817	0.226694	C	1.112825	3.588846	0.247908
C	0.067075	3.886009	-0.864282	C	0.065443	3.896298	-0.842187
H	0.423748	3.593956	-1.858326	H	0.416990	3.599659	-1.836664
H	-0.871155	3.351636	-0.674564	H	-0.877343	3.371295	-0.650317
H	-0.169467	4.956959	-0.894212	H	-0.161002	4.969317	-0.872889
C	2.374105	4.446683	-0.049576	C	2.376183	4.442620	-0.027758
H	2.792730	4.236559	-1.039198	H	2.791494	4.232906	-1.018816
H	2.123242	5.514165	-0.007994	H	2.131362	5.511273	0.017484
H	3.153772	4.249323	0.694612	H	3.155952	4.238593	0.714566
C	0.558219	3.991731	1.603141	C	0.558837	3.994498	1.625940
H	0.267805	5.048864	1.594707	H	0.271475	5.052407	1.619114
H	-0.325919	3.408877	1.879631	H	-0.326579	3.414022	1.903589
H	1.308772	3.859540	2.390735	H	1.310444	3.859668	2.412131

3.1 Å				3.3 Å			
Atom	x	y	z	Atom	x	y	z
Dy	-0.000469	0.000615	-0.437847	Dy	0.000253	-0.000192	-0.399752
Cl	0.000353	-0.000854	-3.506425	Cl	-0.000570	0.000688	-3.718330
C	-2.714929	-0.212950	-0.469303	C	-2.699717	-0.198679	-0.466741
C	-2.351045	-0.017292	0.931255	C	-2.352529	0.001619	0.937240
C	-1.617056	-1.193962	1.317490	C	-1.633563	-1.179647	1.339805
H	-1.280435	-1.397910	2.323578	H	-1.312142	-1.381045	2.351524
C	-1.533998	-2.111579	0.242892	C	-1.545492	-2.104505	0.271695
C	-2.163922	-1.470932	-0.855174	C	-2.152862	-1.463072	-0.839310
H	-2.250893	-1.896261	-1.843826	H	-2.230231	-1.891849	-1.827667
C	-3.612690	0.574223	-1.461164	C	-3.566956	0.596714	-1.478601
C	-5.059843	0.682824	-0.922823	C	-5.018119	0.743613	-0.961106
H	-5.145692	1.292773	-0.022740	H	-5.103769	1.361128	-0.066194
H	-5.462843	-0.310850	-0.695228	H	-5.447804	-0.238909	-0.733719
C	-3.720110	-0.175630	-2.812682	C	-3.672660	-0.164626	-2.823939
H	-4.185267	-1.160877	-2.694527	H	-4.155141	-1.140944	-2.700204
H	-4.357498	0.406948	-3.487680	H	-4.294909	0.421731	-3.509642
H	-2.746001	-0.292980	-3.296144	H	-2.696605	-0.299902	-3.299731
C	-2.892320	0.911164	2.051029	C	-2.894070	0.945775	2.043281
C	-3.169151	2.371461	1.641431	C	-3.138333	2.408127	1.620249
H	-3.958591	2.466526	0.896752	H	-3.911119	2.512746	0.859656
H	-3.488883	2.940619	2.522456	H	-3.464716	2.987717	2.491923
H	-2.271782	2.858061	1.246637	H	-2.225399	2.877195	1.240210
H	-4.019286	-0.726244	2.974718	H	-4.063502	-0.667436	2.956904
C	-1.908880	0.980209	3.246281	C	-1.925478	1.004237	3.251286

H	-0.930121	1.370328	2.950388	H	-0.933478	1.367368	2.964623
H	-2.316476	1.650966	4.011195	H	-2.326893	1.692566	4.003647
H	-1.757607	0.008622	3.725068	H	-1.804350	0.034333	3.742015
C	-1.142118	-3.590821	0.284243	C	-1.183545	-3.590624	0.331934
C	-0.587109	-3.991034	1.663525	C	-0.623272	-3.982367	1.711509
H	-0.313802	-5.052587	1.664726	H	-0.367622	-5.048152	1.723796
H	0.306973	-3.419843	1.932397	H	0.282190	-3.422610	1.965744
H	-1.333217	-3.839931	2.452026	H	-1.359794	-3.809618	2.504585
C	-2.424547	-4.422382	0.027267	C	-2.489125	-4.394477	0.102074
H	-2.840655	-4.217629	-0.964465	H	-2.909164	-4.197149	-0.889507
H	-2.200898	-5.494924	0.086034	H	-2.291650	-5.470962	0.178720
H	-3.195912	-4.192451	0.770804	H	-3.247496	-4.131853	0.848161
C	-0.110018	-3.932530	-0.810096	C	-0.173891	-3.975250	-0.768929
H	-0.464399	-3.644375	-1.805965	H	-0.533480	-3.694943	-1.765098
H	0.844634	-3.423917	-0.634761	H	0.796013	-3.489708	-0.614297
H	0.094104	-5.010212	-0.826823	H	0.000608	-5.058131	-0.770191
H	-5.701852	1.137135	-1.687248	H	-5.637715	1.207289	-1.738083
C	-3.051390	1.974987	-1.795834	C	-2.964735	1.979577	-1.818017
H	-2.086841	1.886987	-2.310943	H	-1.999146	1.861820	-2.326747
H	-3.731147	2.497181	-2.480837	H	-3.622877	2.514610	-2.513990
H	-2.928905	2.605992	-0.914804	H	-2.834646	2.613940	-0.940145
C	-4.205497	0.282807	2.590819	C	-4.225601	0.343540	2.567099
H	-4.972984	0.208839	1.816828	H	-4.983480	0.281412	1.782701
H	-4.608552	0.889598	3.411719	H	-4.628355	0.960615	3.380357
C	2.350627	0.017113	0.931278	C	2.352871	-0.001607	0.937093
C	2.714660	0.212530	-0.469252	C	2.699818	0.198826	-0.466930

C	2.163995	1.470630	-0.855276	C	2.152897	1.463236	-0.839322
H	2.251257	1.895897	-1.843929	H	2.229950	1.892012	-1.827702
C	1.534191	2.111558	0.242698	C	1.545631	2.104529	0.271818
C	1.616887	1.194001	1.317376	C	1.633894	1.179595	1.339835
H	1.280251	1.398129	2.323427	H	1.312615	1.380917	2.351612
C	2.891698	-0.911227	2.051255	C	2.894682	-0.945830	2.042949
C	1.907921	-0.980479	3.246215	C	1.926418	-1.004296	3.251213
H	1.756051	-0.008883	3.724791	H	1.805572	-0.034435	3.742092
H	0.929444	-1.371105	2.950095	H	0.934281	-1.367241	2.964770
H	2.315590	-1.650916	4.011371	H	2.327928	-1.692781	4.003381
C	3.169100	-2.371453	1.641818	C	3.138702	-2.408189	1.619791
H	3.488575	-2.940491	2.523014	H	3.465253	-2.987844	2.491360
H	2.272074	-2.858310	1.246591	H	2.225617	-2.877156	1.239982
H	3.958935	-2.466296	0.897531	H	3.911278	-2.512859	0.858992
C	4.204555	-0.282515	2.591446	C	4.226418	-0.343720	2.566363
H	4.017964	0.726470	2.975337	H	4.064536	0.667283	2.956187
H	4.607533	-0.889234	3.412437	H	4.629357	-0.960812	3.379515
H	4.972255	-0.208299	1.817685	H	4.984066	-0.281687	1.781729
C	3.612917	-0.574529	-1.460753	C	3.566546	-0.596681	-1.479136
C	3.052452	-1.975634	-1.795379	C	2.963806	-1.979362	-1.818378
H	2.929916	-2.606543	-0.914299	H	2.833875	-2.613739	-0.940492
H	2.088046	-1.888187	-2.310808	H	1.998055	-1.861337	-2.326768
H	3.732793	-2.497563	-2.480001	H	3.621482	-2.514568	-2.514660
C	5.059977	-0.682314	-0.921947	C	5.017872	-0.743954	-0.962220
H	5.462396	0.311585	-0.694309	H	5.447895	0.238467	-0.735045
H	5.145840	-1.292147	-0.021782	H	5.103729	-1.361445	-0.067311

H	5.702440	-1.136360	-1.686126	H	5.637046	-1.207810	-1.739419
C	3.720370	0.175106	-2.812401	C	3.671890	0.164697	-2.824476
H	4.185343	1.160458	-2.694397	H	4.154545	1.140938	-2.700842
H	4.357951	-0.407482	-3.487205	H	4.293814	-0.421709	-3.510431
H	2.746282	0.292155	-3.295979	H	2.695693	0.300164	-3.299933
C	1.142681	3.590907	0.283986	C	1.183379	3.590569	0.332096
C	0.110925	3.932954	-0.810585	C	0.173320	3.974895	-0.768489
H	0.465416	3.644655	-1.806375	H	0.532727	3.694710	-1.764756
H	-0.843986	3.424742	-0.635432	H	-0.796395	3.489059	-0.613618
H	-0.092793	5.010710	-0.827392	H	-0.001510	5.057725	-0.769681
C	2.425377	4.422151	0.027357	C	2.488711	4.394701	0.101801
H	2.841644	4.217355	-0.964301	H	2.908568	4.197313	-0.889844
H	2.202011	5.494751	0.086160	H	2.290983	5.471150	0.178306
H	3.196516	4.191944	0.771040	H	3.247323	4.132397	0.847756
C	0.587400	3.991169	1.663147	C	0.623453	3.982264	1.711831
H	0.314162	5.052739	1.664303	H	0.367656	5.048013	1.724163
H	-0.306777	3.420016	1.931809	H	-0.281856	3.422397	1.966366
H	1.333317	3.839994	2.451812	H	1.360247	3.809644	2.504684

3.6 Å				3.8 Å			
Atom	x	y	z	Atom	x	y	z
Dy	0.000430	0.000124	-0.361919	Dy	0.000429	0.000048	-0.323154
Cl	-0.000634	0.008624	-3.930487	Cl	-0.000846	0.009004	-4.141722
C	-2.686261	-0.188412	-0.462567	C	-2.671587	-0.177965	-0.461732
C	-2.355960	0.012910	0.945182	C	-2.360543	0.035917	0.948430
C	-1.649019	-1.172041	1.359757	C	-1.671138	-1.151173	1.386970
H	-1.340762	-1.372827	2.375828	H	-1.378825	-1.343114	2.409537
C	-1.553389	-2.100369	0.295146	C	-1.568789	-2.092612	0.334424
C	-2.140854	-1.456759	-0.825960	C	-2.132239	-1.455159	-0.803176
H	-2.209421	-1.886434	-1.814974	H	-2.195345	-1.896801	-1.787640
C	-3.528410	0.613295	-1.490381	C	-3.471565	0.630715	-1.517018
C	-4.983777	0.783394	-0.992543	C	-4.929069	0.857501	-1.049847
H	-5.073225	1.405143	-0.100855	H	-5.016117	1.492440	-0.167316
H	-5.430693	-0.192095	-0.768040	H	-5.413566	-0.099568	-0.823768
C	-3.625409	-0.153177	-2.833453	C	-3.569482	-0.154521	-2.849219
H	-4.118072	-1.124597	-2.710617	H	-4.082942	-1.113824	-2.716174
H	-4.235427	0.435804	-3.527742	H	-4.160784	0.435894	-3.558170
H	-2.645955	-0.296678	-3.301085	H	-2.589549	-0.321922	-3.308779
C	-2.899271	0.967643	2.040836	C	-2.904690	1.013621	2.022753
C	-3.120021	2.431124	1.608846	C	-3.078373	2.477713	1.571703
H	-3.879464	2.541989	0.835842	H	-3.813160	2.599238	0.776918
H	-3.452918	3.017494	2.473429	H	-3.420038	3.080427	2.421442
H	-2.195954	2.888462	1.241530	H	-2.133999	2.909202	1.225504
H	-4.100542	-0.628233	2.944202	H	-4.166748	-0.544074	2.910770
C	-1.942628	1.018265	3.258729	C	-1.972032	1.051439	3.259632

H	-0.941031	1.360553	2.979084	H	-0.953056	1.352478	2.994769
H	-2.338728	1.719823	4.001516	H	-2.357167	1.779180	3.982560
H	-1.844465	0.050171	3.758150	H	-1.919146	0.088854	3.776374
C	-1.212166	-3.590764	0.367548	C	-1.257004	-3.587981	0.432469
C	-0.651046	-3.978236	1.748007	C	-0.701028	-3.963400	1.818417
H	-0.408499	-5.046900	1.767714	H	-0.478661	-5.035949	1.854944
H	0.262266	-3.427573	1.993984	H	0.222787	-3.426162	2.054400
H	-1.381915	-3.790595	2.542924	H	-1.427003	-3.749815	2.611253
C	-2.532681	-4.374538	0.152063	C	-2.596179	-4.345006	0.234424
H	-2.953305	-4.180730	-0.839959	H	-3.014076	-4.160894	-0.760605
H	-2.353283	-5.453273	0.239900	H	-2.441588	-5.425614	0.343236
H	-3.283258	-4.090846	0.898326	H	-3.338544	-4.029594	0.976169
C	-0.215678	-4.003197	-0.735106	C	-0.274440	-4.043681	-0.665605
H	-0.574620	-3.724816	-1.732015	H	-0.627520	-3.771047	-1.666160
H	0.764490	-3.536197	-0.589394	H	0.718729	-3.602595	-0.528883
H	-0.063375	-5.089290	-0.728010	H	-0.153254	-5.133339	-0.642160
H	-5.585757	1.252804	-1.779662	H	-5.498603	1.337095	-1.854580
C	-2.899184	1.984752	-1.828064	C	-2.785238	1.972939	-1.862394
H	-1.932891	1.849866	-2.332523	H	-1.813782	1.795619	-2.346177
H	-3.541660	2.528941	-2.531366	H	-3.389027	2.527845	-2.590969
H	-2.765668	2.619728	-0.950977	H	-2.650678	2.618008	-0.992501
C	-4.244331	0.383988	2.550450	C	-4.274834	0.468808	2.507410
H	-4.993495	0.330514	1.757056	H	-5.007996	0.429443	1.698414
H	-4.648278	1.008187	3.357581	H	-4.679169	1.109123	3.301572
C	2.356490	-0.015041	0.944967	C	2.361207	-0.038130	0.948167
C	2.686685	0.188988	-0.462416	C	2.672086	0.178759	-0.461582

C	2.141547	1.458117	-0.823317	C	2.132763	1.456655	-0.800264
H	2.209679	1.889528	-1.811600	H	2.195497	1.900282	-1.783856
C	1.554172	2.099590	0.299033	C	1.569316	2.091652	0.338703
C	1.649705	1.169221	1.361864	C	1.671912	1.148043	1.389293
H	1.341632	1.368064	2.378362	H	1.379653	1.337704	2.412289
C	2.900283	-0.971752	2.038646	C	2.905541	-1.018221	2.020205
C	1.944534	-1.024045	3.257174	C	1.973763	-1.057902	3.257693
H	1.846971	-0.056687	3.758143	H	1.921785	-0.096269	3.776306
H	0.942636	-1.365698	2.977800	H	0.954412	-1.357905	2.993060
H	2.341043	-1.726816	3.998595	H	2.359052	-1.787227	3.978938
C	3.120119	-2.434612	1.604052	C	3.077896	-2.481535	1.566127
H	3.453190	-3.022626	2.467450	H	3.419666	-3.086230	2.414415
H	2.195643	-2.890890	1.236426	H	2.132968	-2.911606	1.219666
H	3.879096	-2.544507	0.830456	H	3.812072	-2.601970	0.770613
C	4.245953	-0.389441	2.548151	C	4.276379	-0.475247	2.504948
H	4.102839	0.622167	2.943717	H	4.169238	0.536895	2.910413
H	4.650368	-1.015122	3.353903	H	4.680904	-1.117392	3.297535
H	4.994464	-0.334888	1.754215	H	5.008957	-0.434724	1.695484
C	3.527493	-0.611494	-1.492276	C	3.470906	-0.628260	-1.519010
C	2.896476	-1.981651	-1.831858	C	2.782898	-1.968998	-1.866781
H	2.763286	-2.618164	-0.955830	H	2.648886	-2.616061	-0.998287
H	1.929755	-1.844869	-2.335081	H	1.810938	-1.789647	-2.348891
H	3.537450	-2.525233	-2.537004	H	3.385069	-2.522726	-2.597590
C	4.983106	-0.784007	-0.995998	C	4.928448	-0.857624	-1.053267
H	5.431334	0.190688	-0.770665	H	5.414178	0.098495	-0.825820
H	5.072749	-1.406980	-0.105184	H	5.015432	-1.494228	-0.171931

H	5.583835	-1.253051	-1.784301	H	5.496887	-1.336399	-1.859265
C	3.624183	0.157381	-2.833998	C	3.568859	0.159831	-2.849532
H	4.117605	1.128207	-2.709602	H	4.082969	1.118496	-2.714466
H	4.233374	-0.430734	-3.529751	H	4.159579	-0.429380	-3.559972
H	2.644563	0.302590	-3.300777	H	2.588901	0.328998	-3.308421
C	1.211983	3.589627	0.373833	C	1.256831	3.586665	0.439599
C	0.214585	4.002874	-0.727696	C	0.273858	4.043773	-0.657535
H	0.573032	3.725903	-1.725174	H	0.626921	3.773089	-1.658621
H	-0.765229	3.535132	-0.581996	H	-0.719047	3.601876	-0.521476
H	0.061586	5.088863	-0.719138	H	0.152032	5.133317	-0.632129
C	2.531796	4.374671	0.158676	C	2.595574	4.344758	0.242739
H	2.952094	4.182453	-0.833794	H	3.013472	4.162650	-0.752661
H	2.351624	5.453157	0.247945	H	2.440425	5.425091	0.353472
H	3.282949	4.090622	0.904227	H	3.338187	4.028440	0.983853
C	0.651420	3.974732	1.755183	C	0.700851	3.959240	1.826306
H	0.408094	5.043186	1.776478	H	0.477947	5.031607	1.864824
H	-0.261341	3.423078	2.001013	H	-0.222669	3.421108	2.061424
H	1.382933	3.786568	2.549389	H	1.427026	3.744576	2.618674

4.1 Å			
Atom	x	y	z
Dy	-0.007517	-0.007668	-0.278249
Cl	-0.005274	-0.236903	-4.340364
C	-2.663520	-0.177624	-0.456141
C	-2.376810	0.093843	0.949270
C	-1.709602	-1.080372	1.451817
H	-1.436576	-1.229289	2.486737
C	-1.595695	-2.067003	0.442218
C	-2.132392	-1.473757	-0.732279
H	-2.184133	-1.956232	-1.698519
C	-3.405291	0.607789	-1.569496
C	-4.859559	0.930548	-1.153137
H	-4.936125	1.611827	-0.305131
H	-5.397909	0.010925	-0.895630
C	-3.511202	-0.240589	-2.862085
H	-4.059775	-1.173432	-2.686990
H	-4.072086	0.332790	-3.608661
H	-2.533020	-0.463495	-3.303110
C	-2.922126	1.130492	1.965436
C	-3.019788	2.580550	1.451045
H	-3.710725	2.697438	0.617299
H	-3.375642	3.230490	2.259107
H	-2.043675	2.960265	1.133903
H	-4.274336	-0.343873	2.863390
C	-2.031235	1.182036	3.232467

H	-0.990302	1.424246	2.991804
H	-2.406074	1.958325	3.908677
H	-2.041550	0.242021	3.791849
C	-1.303170	-3.559670	0.611145
C	-0.741870	-3.875789	2.009512
H	-0.537217	-4.948869	2.097016
H	0.192878	-3.343391	2.211218
H	-1.457110	-3.611733	2.796897
C	-2.655278	-4.304810	0.458860
H	-3.079607	-4.159539	-0.539954
H	-2.515552	-5.381607	0.615040
H	-3.386031	-3.945354	1.192097
C	-0.336211	-4.082008	-0.471031
H	-0.692590	-3.852696	-1.481175
H	0.664453	-3.649997	-0.363438
H	-0.230972	-5.170853	-0.395777
H	-5.383162	1.397760	-1.995283
C	-2.640024	1.893928	-1.960160
H	-1.674863	1.643353	-2.426291
H	-3.198240	2.452209	-2.721612
H	-2.480015	2.565740	-1.114570
C	-4.328106	0.657126	2.421189
H	-5.035966	0.616758	1.590118
H	-4.731908	1.341055	3.178378
C	2.365318	-0.031454	0.949008
C	2.650858	0.156245	-0.470256

C	2.116992	1.433362	-0.822160
H	2.173887	1.860239	-1.813915
C	1.583471	2.095933	0.316251
C	1.695528	1.169603	1.381283
H	1.424884	1.382071	2.405923
C	2.916497	-0.999266	2.028312
C	2.007271	-0.998010	3.283165
H	1.994402	-0.029666	3.791690
H	0.974263	-1.268199	3.038874
H	2.382407	-1.731391	4.005517
C	3.057793	-2.473512	1.600013
H	3.417290	-3.064631	2.450490
H	2.098591	-2.899931	1.290355
H	3.766664	-2.618996	0.786004
C	4.305462	-0.468878	2.473776
H	4.223706	0.553903	2.857850
H	4.714676	-1.099915	3.272802
H	5.022586	-0.459115	1.649552
C	3.411037	-0.683080	-1.530889
C	2.688481	-2.015778	-1.839185
H	2.541440	-2.635123	-0.952451
H	1.721321	-1.828046	-2.329113
H	3.274170	-2.604461	-2.555733
C	4.873715	-0.931245	-1.091622
H	5.380738	0.019425	-0.888963
H	4.968106	-1.556181	-0.202677

H	5.416339	-1.431978	-1.901857
C	3.493365	0.079392	-2.877243
H	4.023232	1.033150	-2.770024
H	4.061682	-0.531081	-3.587783
H	2.507939	0.249995	-3.324844
C	1.309166	3.599621	0.403234
C	0.339759	4.076645	-0.697374
H	0.682217	3.784721	-1.696171
H	-0.666881	3.669680	-0.555391
H	0.254884	5.169779	-0.683038
C	2.669445	4.317086	0.198287
H	3.080305	4.113939	-0.795951
H	2.545120	5.402256	0.299721
H	3.403774	3.986402	0.941358
C	0.766409	4.001499	1.787250
H	0.574916	5.080212	1.815387
H	-0.171885	3.493197	2.029615
H	1.487773	3.774209	2.580394

References

(S1) Dehmlow;, E. V.; Bollmann, C. Verbesserte präparative Darstellung von Polyisopropyl- und Poly-terf-butylcyclopentadienen. *Z. Naturforsch. B* **1993**, *48b*, 457–460.

(S2) Evans, W. J.; Kozimor, S. A.; Ziller, J. W.; Fagin, A. A.; Bochkarev, M. N. Facile Syntheses of Unsolvated UI_3 and Tetramethylcyclopentadienyl Uranium Halides. *Inorg. Chem.* **2005**, *44*, 3993–4000.

(S3) Goodwin, C. A. P.; Ortu, F.; Reta, D.; Chilton, N. F.; Mills, D. P. Molecular magnetic hysteresis at 60 kelvin in dysprosocenium. *Nature* **2017**, *548*, 439–442.

(S4) Lindley, B. M.; Jacobs, B. P.; MacMillan, S. N.; Wolczanski, P. T. Neutral Fe(IV) alkylidenes, including some that bind dinitrogen. *Chem. Commun.* **2016**, *52*, 3891–3894.

(S5) Bodach, A.; Bamford, K. L.; Longobardi, L. E.; Felderhoff, M.; Stephan, D. W. Group 13-derived radicals from α-diimines via hydro- and carboalumination reactions. *Dalton Trans.* **2020**, *49*, 11689–11696.

(S6) Korzyński, M. D.; Berkson, Z. J.; Le Guennic, B.; Cador, O.; Copéret, C. Leveraging Surface Siloxide Electronics to Enhance the Relaxation Properties of a Single-Molecule Magnet. *J. Am. Chem. Soc.* **2021**, *143*, 5438–5444.

(S7) Fung, B. M.; Khitrin, A. K.; Ermolaev, K. An Improved Broadband Decoupling Sequence for Liquid Crystals and Solids. *J. Magn. Reson.* **2000**, *142*, 97–101.

(S8) Hung, I.; Rossini, A. J.; Schurko, R. W. Application of the Carr-Purcell Meiboom-Gill Pulse Sequence for the Acquisition of Solid-State NMR Spectra of Spin-1/2 Nuclei. *J. Phys. Chem. A* **2004**, *108*, 7112–7120.

(S9) Zagdoun, A.; Casano, G.; Ouari, O.; Schwarzwälder, M.; Rossini, A. J.; Aussenac, F.; Yulikov, M.; Jeschke, G.; Copéret, C.; Lesage, A.; Tordo, P.; Emsley, L. Large Molecular Weight Nitroxide Biradicals Providing Efficient Dynamic Nuclear Polarization at Temperatures up to 200 K. *J. Am. Chem. Soc.* **2013**, *135*, 12790–12797.

(S10) O'Dell, L. A.; Schurko, R. W. QCPMG using adiabatic pulses for faster acquisition of ultra-wideline NMR spectra. *Chem. Phys. Lett.* **2008**, *464*, 97–102.

(S11) O'Dell, L. A. *Modern Magnetic Resonance*; Springer International Publishing, 2017.

(S12) Lokshin, B. V.; Aleksanyan, V. T.; Ezernitskaya, M. G. Vibrational spectra of mesitylene. *Russ. Chem. Bull.* **1982**, *31*, 1995–1999.

(S13) Copéret, C.; Comas-Vives, A.; Conley, M. P.; Estes, D. P.; Fedorov, A.; Mougel, V.; Nagae, H.; Núnez-Zarur, F.; Zhizhko, P. A. Surface Organometallic and Coordination Chemistry toward Single-Site Heterogeneous Catalysts: Strategies, Methods, Structures, and Activities. *Chem. Rev.* **2016**, *116*, 323–421.

(S14) Protsak, I. S.; Morozov, Y. M.; Dong, W.; Le, Z.; Zhang, D.; Henderson, I. M. A ^{29}Si, ^{1}H, and ^{13}C Solid-State NMR Study on the Surface Species of Various Depolymerized Organosiloxanes at Silica Surface. *Nanoscale Res. Lett.* **2019**, *160*, 1–15.

(S15) Sanchez Escribano, V.; Garbarino, G.; Finocchio, E.; Busca, G. y-Alumina and Amorphous Silica–Alumina: Structural Features, Acid Sites and the Role of Adsorbed Water. *Top. Catal.* **2017**, *60*, 1554–1564.

(S16) Barzetti, T.; Selli, E.; Moscotti, D.; Forni, L. Pyridine and ammonia as probes for FTIR analysis of solid acid catalysts. *J. Chem. Soc., Faraday Trans.* **1996**, *92*, 1401–1407.

(S17) Moroz, I. B.; Larmier, K.; Liao, W.-C.; Copéret, C. Discerning γ-Alumina Surface Sites

with Nitrogen-15 Dynamic Nuclear Polarization Surface Enhanced NMR Spectroscopy of Adsorbed Pyridine. *J. Phys. Chem. C* **2018**, *122*, 10871–10882.

(S18) Brinkmann, A.; Kentgens, A. P. M. Proton-Selective ^{17}O-^{1}H Distance Measurements in Fast Magic-Angle-Spinning Solid-State NMR Spectroscopy for the Determination of Hydrogen Bond Lengths. *J. Am. Chem. Soc.* **2006**, *128*, 14758–14759.

(S19) Liao, W.-C.; Ghaffari, B.; Gordon, C. P.; Xu, J.; Copéret, C. Dynamic Nuclear Polarization Surface Enhanced NMR spectroscopy (DNP SENS): Principles, protocols, and practice. *Curr. Opin. Colloid Interface Sci.* **2018**, *33*, 63–71.

(S20) Rossini, A. J.; Zagdoun, A.; Lelli, M.; Lesage, A.; Copéret, C.; Emsley, L. Dynamic Nuclear Polarization Surface Enhanced NMR Spectroscopy. *Acc. Chem. Res.* **2013**, *46*, 1942–1951.

(S21) Pascal, P.; Pacault, A.; Hoarau, J. Contribution a Letude dune sytematique magnetochimique. *C. R. Acad. Sci.* **1951**, *233*, 1078–1080.

(S22) Bain, G. A.; Berry, J. F. Diamagnetic corrections and Pascal's constants. *J. Chem. Educ.* **2008**, *85*, 532–536.

(S23) McClain, K. R.; Gould, C. A.; Chakarawet, K.; Teat, S. J.; Groshens, T. J.; Long, J. R.; Harvey, B. G. High-temperature magnetic blocking and magneto-structural correlations in a series of dysprosium(III) metallocenium single-molecule magnets. *Chem. Sci.* **2018**, *9*, 8492–8503.

(S24) Gould, C. A.; McClain, K. R.; Yu, J. M.; Groshens, T. J.; Furche, F.; Harvey, B. G.; Long, J. R. Synthesis and Magnetism of Neutral, Linear Metallocene Complexes of Terbium(II) and Dysprosium(II). *J. Am. Chem. Soc.* **2019**, *141*, 12967–12973.

(S25) Frisch, M. J.; Trucks, G. W.; Schlegel, H. B.; Scuseria, G. E.; Robb, M. A.; Cheeseman, J. R.; Scalmani, G.; Barone, V.; Mennucci, B.; Petersson, G. A.; Nakatsuji, H.;

Caricato, M.; Li, X.; Hratchian, H. P.; Izmaylov, J., A. F.and Bloino; Zheng, G.; Sonnenberg, J. L.; Hada, M.; Ehara, M.; Toyota, K.; Fukuda, R.; Hasegawa, J.; Ishida, M.; Nakajima, T.; Honda, Y.; Kitao, O.; Nakai, H.; Vreven, T.; Montgomery Jr., J. A.; Peralta, J. E.; Ogliaro, F.; Bearpark, M.; Heyd, E., J. J.and Brothers; Kudin, K. N.; Staroverov, T., V. N.and Keith; Kobayashi, R.; Normand, J.; Raghavachari, K.; Rendell, A.; Burant, J. C.; Iyengar, S. S.; Tomasi, J.; Cossi, M.; Rega, N.; Millam, J. M.; Klene, M.; Knox, J. E.; Cross, J. B.; Bakken, V.; Adamo, C.; Jaramillo, J.; Gomperts, R.; Stratmann, R. E.; Yazyev, O.; Austin, A. J.; Cammi, C., R.and Pomelli; Ochterski, J. W.; Martin, R. L.; Morokuma, K.; Zakrzewski, V. G.; Voth, G. A.; Salvador, P.; Dannenberg, J. J.; Dapprich, S.; Daniels, A. D.; Farkas, O.; Foresman, J. B.; Ortiz, J. V.; Cioslowski, J.; Fox, D. J. *Gaussian 09, Revision D.01* **Gaussian, Inc., Wallingford CT**, 2013.

(S26) Becke, A. D. Density-functional thermochemistry. III. The role of exact exchange. *J. Chem. Phys.* **1993**, *98*, 5648–5652.

(S27) Hehre, W. J.; Ditchfield, R.; Pople, J. A. Self—Consistent Molecular Orbital Methods. XII. Further Extensions of Gaussian—Type Basis Sets for Use in Molecular Orbital Studies of Organic Molecules. *J. Chem. Phys.* **1972**, *56*, 2257–2261.

(S28) Roy, L. E.; Hay, P. J.; Martin, R. L. Revised Basis Sets for the LANL Effective Core Potentials. *J. Chem. Theory Comput.* **2008**, *4*, 1029–1031.

(S29) Fdez Galván, I.; Vacher, M.; Alavi, A.; Angeli, C.; Aquilante, F.; Autschbach, J.; Bao, J. J.; Bokarev, S. I.; Bogdanov, N. A.; Carlson, R. K.; Chibotaru, L. F.; Creutzberg, J.; Dattani, N.; Delcey, M. G.; Dong, S. S.; Dreuw, A.; Freitag, L.; Frutos, L. M.; Gagliardi, L.; Gendron, F.; Giussani, A.; González, L.; Grell, G.; Guo, M.; Hoyer, C. E.; Johansson, M.; Keller, S.; Knecht, S.; Kovačević, G.; Källman, E.; Li Manni, G.; Lundberg, M.; Ma, Y.; Mai, S.; Malhado, J. P.; Malmqvist, P. A.; Marquetand, P.; Mewes, S. A.; Norell, J.; Olivucci, M.; Oppel, M.; Phung, Q. M.; Pier-

loot, K.; Plasser, F.; Reiher, M.; Sand, A. M.; Schapiro, I.; Sharma, P.; Stein, C. J.; Sørensen, L. K.; Truhlar, D. G.; Ugandi, M.; Ungur, L.; Valentini, A.; Vancoillie, S.; Veryazov, V.; Weser, O.; Wesołowski, T. A.; Widmark, P. O.; Wouters, S.; Zech, A.; Zobel, J. P.; Lindh, R. OpenMolcas: From Source Code to Insight. *J. Chem. Theory. Comput.* **2019**, *15*, 5925–5964.

(S30) Roos, B. O.; Taylor, P. R.; Siegbahn, P. E. M. A Complete Active Space Scf Method (Casscf) Using a Density-Matrix Formulated Super-Ci Approach. *Chem. Phys.* **1980**, *48*, 157–173.

(S31) Malmqvist, P. A.; Roos, B. O. The Casscf State Interaction Method. *Chem. Phys. Lett.* **1989**, *155*, 189–194.

(S32) Malmqvist, P. A.; Roos, B. O.; Schimmelpfennig, B. The restricted active space (RAS) state interaction approach with spin-orbit coupling. *Chem. Phys. Lett.* **2002**, *357*, 230–240.

(S33) Roos, B. O.; Lindh, R.; Malmqvist, P. A.; Veryazov, V.; Widmark, P. O. Main group atoms and dimers studied with a new relativistic ANO basis set. *J. Phys. Chem. A* **2004**, *108*, 2851–2858.

(S34) Roos, B. O.; Lindh, R.; Malmqvist, P. A.; Veryazov, V.; Widmark, P. O.; Borin, A. C. New relativistic atomic natural orbital basis sets for lanthanide atoms with applications to the Ce diatom and LuF_3. *J. Phys. Chem. A* **2008**, *112*, 11431–11435.

(S35) Aquilante, F.; Malmqvist, P. A.; Pedersen, T. B.; Ghosh, A.; Roos, B. O. Cholesky Decomposition-Based Multiconfiguration Second-Order Perturbation Theory (CD-CASPT2): Application to the Spin-State Energetics of Co(III)(diiminato)(NPh). *J. Chem. Theory. Comput.* **2008**, *4*, 694–702.

(S36) Chibotaru, L. F.; Ungur, L. Ab initio calculation of anisotropic magnetic properties

of complexes. I. Unique definition of pseudospin Hamiltonians and their derivation. *J. Chem. Phys.* **2012**, *137*, 064112.
